# Evolutionary Maintenance of the PTS2 Protein Import Pathway in the Stramenopile Alga *Nannochloropsis*

**DOI:** 10.3389/fcell.2020.593922

**Published:** 2020-11-19

**Authors:** Dmitry Kechasov, Imke de Grahl, Pierre Endries, Sigrun Reumann

**Affiliations:** ^1^Centre for Organelle Research, University of Stavanger, Stavanger, Norway; ^2^Plant Biochemistry and Infection Biology, Institute for Plant Science and Microbiology, Universität Hamburg, Hamburg, Germany

**Keywords:** peroxisome biogenesis, microalgae (unicellular eukaryotic algae), evolution, malate synthase, thiolase, PTS2 protein import pathway

## Abstract

The stramenopile alga *Nannochloropsis* evolved by secondary endosymbiosis of a red alga by a heterotrophic host cell and emerged as a promising organism for biotechnological applications, such as the production of polyunsaturated fatty acids and biodiesel. Peroxisomes play major roles in fatty acid metabolism but experimental analyses of peroxisome biogenesis and metabolism in *Nannochloropsis* are not reported yet. In fungi, animals, and land plants, soluble proteins of peroxisomes are targeted to the matrix by one of two peroxisome targeting signals (type 1, PTS1, or type 2, PTS2), which are generally conserved across kingdoms and allow the prediction of peroxisomal matrix proteins from nuclear genome sequences. Because diatoms lost the PTS2 pathway secondarily, we investigated its presence in the stramenopile sister group of diatoms, the *Eustigmatophyceae*, represented by *Nannochloropsis*. We detected a full-length gene of a putative *PEX7* ortholog coding for the cytosolic receptor of PTS2 proteins and demonstrated its expression in *Nannochloropsis gaditana*. The search for predicted PTS2 cargo proteins in *N. gaditana* yielded several candidates. *In vivo* subcellular targeting analyses of representative fusion proteins in different plant expression systems demonstrated that two predicted PTS2 domains were indeed functional and sufficient to direct a reporter protein to peroxisomes. Peroxisome targeting of the predicted PTS2 cargo proteins was further confirmed in *Nannochloropsis oceanica* by confocal and transmission electron microscopy. Taken together, the results demonstrate for the first time that one group of stramenopile algae maintained the import pathway for PTS2 cargo proteins. To comprehensively map and model the metabolic capabilities of *Nannochloropsis* peroxisomes, *in silico* predictions needs to encompass both the PTS1 and the PTS2 matrix proteome.

## Introduction

Peroxisomes are small organelles of 0.1–1.7 μm in diameter, surrounded by a single membrane and able to detoxify reactive oxygen species generated by diverse peroxisomal enzymes. Common peroxisomal functions of animals and plants are the glyoxylate cycle, amino acid metabolism, and polyamine oxidation. Plant peroxisomes are furthermore capable of contributing to photorespiration and to several biosynthetic functions, such as jasmonate, phylloquinone, and biotin biosynthesis (Reumann and Bartel, 2016; Pan et al., 2020). Importantly, peroxisomes play vital roles in lipid metabolism in many organisms. While in animals fatty acid β-oxidation occurs in both mitochondria and peroxisomes (Wanders and Waterham, 2006), in plants the process takes place exclusively in peroxisomes (Graham and Eastmond, 2002). Hence, peroxisomal metabolism determines the steady state levels of total cellular fatty acids. In mammals, peroxisomes are additionally involved in the biosynthesis of docosahexaenoic acid (C22:6n-3) and eicosapentaenoic acid (EPA; C20:5n-3) by partial degradation of polyunsaturated fatty acids (PUFAs) with longer chains, such as C24:6n-3 (Sprecher, 2000). Knowledge of the metabolic capabilities of peroxisomes from plants, yeast, and fungi in lipid metabolism is prerequisite for successful biotechnological applications and genetic engineering to increase PUFA and biodiesel productivity.

All peroxisomal matrix proteins are encoded in the nuclear genome, synthesized on cytosolic ribosomes and are transported into peroxisomes with the help of peroxins (PEX proteins). Matrix proteins are targeted to peroxisomes by two major import pathways, depending on the possession of two distinct signals, called the peroxisomal targeting signal type 1 (PTS1) or type 2 (PTS2) (Gould et al., 1989; Swinkels et al., 1992). The PTS1 pathway predominates in all organisms studied to date and relies on the presence of a conserved C-terminal tripeptide. The twelve PTS1 tripeptides included in the plant consensus sequence [SA]-[KR]-[LMI]> have the highest peroxisomal targeting probability, but many additional non-canonical tripeptides have been characterized particularly in plants (Lingner et al., 2011). Additional residues upstream of the C-terminal tripeptide often contribute to peroxisome targeting (Brocard and Hartig, 2006; Lingner et al., 2011). Generally, the PTS2 nonapeptide is located within the first 40 amino acid (aa) residues and is cleaved upon transport into peroxisomes by the DEG15 protease (Helm et al., 2007; Schuhmann et al., 2008). Four amino acid residues are most conserved in canonical PTS2 nonapeptides included in the motif [RK]-[LVI]-x_5_-[HQ]-[LAF], where x_5_ denotes four variable amino acid residues and one hydrophobic residue in the middle (Osumi et al., 1992; Swinkels et al., 1992; Petriv et al., 2004; Kunze et al., 2011; Kunze, 2020). Two cytosolic receptors, PEX5 and PEX7, recognize the PTS1 and PTS2 of soluble proteins, respectively, and transport them to the peroxisomal membrane (Kragler et al., 1998; Gatto et al., 2000). PEX5 possesses at least two functional domains, namely the C-terminal half with seven tetratricopeptide (34-amino acid) repeats, which form the PTS1 binding pocket, and the N-terminal half bearing several diaromatic WxxxF/Y motifs that bind PEX14. PEX5 homologs of basidiomycetes, animals and plants additionally contain a PEX7 binding domain of approximately 37 amino acid residues in the N-terminal domain and also function as PEX7 co-receptors (Dodt et al., 2001; Woodward and Bartel, 2005). The docking complex proteins, PEX13 and PEX14, are responsible for initial PEX5-PEX7-cargo binding to the peroxisomal membrane (Bartel et al., 2014).

Peroxisomes are present in nearly all eukaryotes (Tolbert and Essner, 1981; Gabaldón, 2010). Particularly in eukaryotic microorganisms, such as algae and protists, basic research data are scarce regarding the biogenesis mechanisms and physiological functions of peroxisomes. In *Closterium ehrenbergii*, a charophyte alga of the family of *Zygnematophyceae*, peroxisomes have been visualized by H_2_O_2_/diaminobenzidine staining (Shinozaki et al., 2009; Hayashi and Shinozaki, 2012). In the chlorophyte alga *Chlamydomonas*, all enzymes associated with the glyoxylate cycle except for isocitrate lyase were located in punctate structures indicative of peroxisomes (Lauersen et al., 2016). In chromalveolates (organisms containing complex plastids of red algal ancestry), peroxisomes have mainly been studied in diatoms and alveolates (Gonzalez et al., 2011; Moog et al., 2017; Ludewig-Klingner et al., 2018). Alveolates, which include apicomplexa, dinoflagellates and ciliates, were long thought to lack peroxisomes, but coccidian apicomplexa, such as *Toxoplasma*, have recently been shown to possess peroxisomes, while other groups (e.g., *Plasmodium*) indeed lack this organelle (Moog et al., 2017). In the cryptophyte *Guillardia theta*, which possesses two phylogenetically different nuclei of host and endosymbiotic origin, a complete set of peroxins including PEX7 was identified and some of them were heterologously localized in peroxisomes as green fluorescent protein (GFP) fusions in *Phaeodactylum tricornutum* (Mix et al., 2018). In the same study, genome analyses indicated the presence of peroxins and, hence, peroxisomes in three stramenopile algae (*Nannochloropsis gaditana*, *Aureococcus anophagefferens*, and the brown alga, *Ectocarpus siliculosus*). In contrast, two haptophytes (*Emiliania huxleyi* and *Chrysochromulina tobin*) appeared to lack essential peroxins (Mix et al., 2018). Experimental analyses of peroxisomes in *Nannochloropsis* by microscopy or biochemistry have not been reported to date.

While the PTS1 targeting pathway is ubiquitous in all organisms that possess peroxisomes, the PTS2 targeting pathway is absent in specific organisms, such as *Caenorhabditis elegans* (Motley et al., 2000), *Drosophila melanogaster* (Faust et al., 2012; Baron et al., 2016) and few microalgae. As revealed by genome sequencing of the red alga *Cyanidioschyzon merolae*, the PEX7 receptor and PTS2 containing cargo proteins are absent (Matsuzaki et al., 2004; Shinozaki et al., 2009). The most comprehensive studies have been reported for the stramenopile diatom, *P. tricornutum*, which accordingly lacks PEX7 and predicted PTS2 proteins and is not capable of importing foreign PTS2 cargo proteins into the peroxisomal matrix (Gonzalez et al., 2011).

Stramenopiles (or heterokonts) form a large and diverse subgroup of chromalveolates that possess two morphologically different flagella and share the same evolutionary history of secondary endosymbiosis between a eukaryotic red alga and a heterotrophic eukaryotic host cell. Stramenopiles include both photosynthetic members (the ochrophytes) with complex plastids as well as aplastidic, non-photosynthetic members (e.g., oomycetes, Supplementary Figure 1). As a relic of evolution and phagocytosis, the plastids of photosynthetic stramenopiles are often still surrounded by four membranes (McFadden, 2001; Barsanti and Gualtieri, 2014). The photosynthetic group of stramenopiles includes many ecologically important lineages (diatoms, brown algae, pelagophytes) as well as the *Eustigmatophyceae* (e.g., *Nannochloropsis*) and forms the most significant component of eukaryotic marine phytoplankton (Guiry, 2012; Qiu et al., 2013; Dorrell et al., 2017).

*Nannochloropsis* is the best known representative of the *Eustigmatophyceae* and recently attracted increasing attention due to its high lipid accumulation of up to 60% of biomass dry weight (Rodolfi et al., 2009) and its outstanding EPA productivity [up to 4.3% (w/w) of biomass dry weight, Camacho-Rodriguez et al., 2014]. Therefore, *Nannochloropsis* species are considered promising candidates for the production of algal biomass and lipids for biofuels and high-value products (Ma et al., 2016). The genome sequences of the first *Nannochloropsis* strains have been published, including *N. gaditana* CCMP526 (Radakovits et al., 2012), *N. gaditana* B-31 (Corteggiani Carpinelli et al., 2014), and *N. oceanica* CCMP1779 and IMET-1 (Vieler et al., 2012; Wang et al., 2014). *In silico* analyses indicated the presence of genes encoding a putative PEX5 ortholog and putative PTS1 proteins. Due to the close phylogenetic relationship between red algae, diatoms and *Eustigmatophyceae*, the absence of the PTS2 pathway in *Nannochloropsis* was considered likely (Lauersen et al., 2016). In fact, not any PTS2 containing protein has been identified computationally or experimentally in this genus to date despite the availability of sequencing data for several *Nannochloropsis* species.

In the present study, we verified the expression of a putative *PEX7* gene from *N. gaditana*. By comprehensive computational analyses, we predicted putative PTS2 cargo proteins in *Nannochloropsis* and identified more than a dozen of candidate proteins. We demonstrated that several PTS2-carrying cargo proteins of *N. gaditana* were indeed imported into peroxisomes in two plant expression systems and also in *Nannochloropsis* itself. Taken together, the results demonstrate for the first time experimentally that this stramenopile alga has maintained the PTS2 protein import pathway, even though the same trafficking route has been lost secondarily in the stramenopile sister group of diatoms.

## Materials and Methods

### Computational Analyses

For the identification of putative orthologs of *Arabidopsis* PEX and PTS2 proteins, the *Arabidopsis* proteins were used as queries in homology searches (BLASTp at NCBI, BLOSUM62 matrix, and standard parameters, McGinnis and Madden, 2004; Boratyn et al., 2013) against the non-redundant database of proteins of *N. gaditana* strains B-31 and CCMP526. Proteins with significant similarity (*E*-value <0.001, >30% identity, >50% of query length) were analyzed for the presence of a predicted PTS1 (PredPlantPTS1, Reumann et al., 2012) and a PTS2 included in the motif [RK]-[LVI]-x_5_-[HQ]-[LAF] (manual analyses, Kunze et al., 2011).

To identify yet unknown PTS2 proteins, the predicted protein sequences of *N. gaditana* B-31 (version 1.0, accessed on October 2, 2014) and CCMP526 (version 1.1, accessed on July 14, 2014) were downloaded. The sequences were analyzed for canonical PTS2 nonapeptides (see above) by applying the search algorithm “advanced find” of Microsoft Word. In *N. gaditana* B-31 more than 50 proteins were found, 13 of which contained the PTS2 motif in the N-terminal 40-amino acid domain (Table 1). For analyses of PTS2 conservation, homologous sequences were aligned by COBALT (Papadopoulos and Agarwala, 2007) and sequence conservation labeled by Boxshade.

**TABLE 1:** Predicted PTS2 proteins of *N. gaditana.*

Acc. number	Annotation	Acronym	Predicted PTS2 (PTS1)	
			Nonapeptide sequence	Position
**Homologs of known *Arabidopsis* PTS2 proteins**				
EWM30341.1	Malate synthase 2	NgMLS2	RIx_5_HL	10–18
EWM24705.1	Peroxisomal 3-ketoacyl-CoA thiolase	NgPKT	RLx_5_HL	12–20
EWM27800.1	Transthyretin family protein	NgHIUase	RLx_5_HL	12–20
EWM29206.1	Protein kinase c binding protein / histidine triad family protein 1	NgHIT1	RLx_5_HL	4–12
**Putative novel *Nannochloropsis* PTS2 proteins**				
EWM21473.1	Embryogenesis-associated protein EMB8 / α/β hydrolase		RLx_5_QL (SRL>)	35–43
EWM25437.1	Vacuolar transporter chaperone 4		KLx_5_QL	24–32
EWM24223.1	Aldo/keto reductase		RIx_5_HL	16–24
EWM30441.1	Didehydrogluconate reductase		RVx_5_HA	19–27
EWM20598.1	Acetyl-/succinylornithine aminotransferase		RLx_5_QA	29–37
EWM21839.1	Protein polybromo-1		KLx_5_QL	2–10
EWM28361.1	Histone H4 acetyltransferase, NuA4 complex, Eaf6		KVx_5_HA	10–18
EWM21222.1	Hypothetical protein		RLx_5_QL	20–28
EWM27137.1	Hypothetical protein		RLx_5_HL	6–14

*The predicted protein sequences of *N. gaditana* B-31 and CCMP526 were analyzed for predicted PTS2 proteins using the canonical PTS2 motif [RK]-[LVI]-x_5_-[HQ]-[LAF] (Kunze et al., 2011) converted to a regular expression. Protein annotations are provided according to GenBank flat files (NCBI, Benson et al., 2005). Thirteen proteins with predicted PTS2 in the N-terminal 40-aa domain were detected in *N. gaditana* strain B-31, all of which were also detected as putative PTS2 proteins in strain CCMP526. One predicted PTS2 protein (EWM21473.1) additionally contained a predicted PTS1 (SRL>).*

**FIGURE 1:**
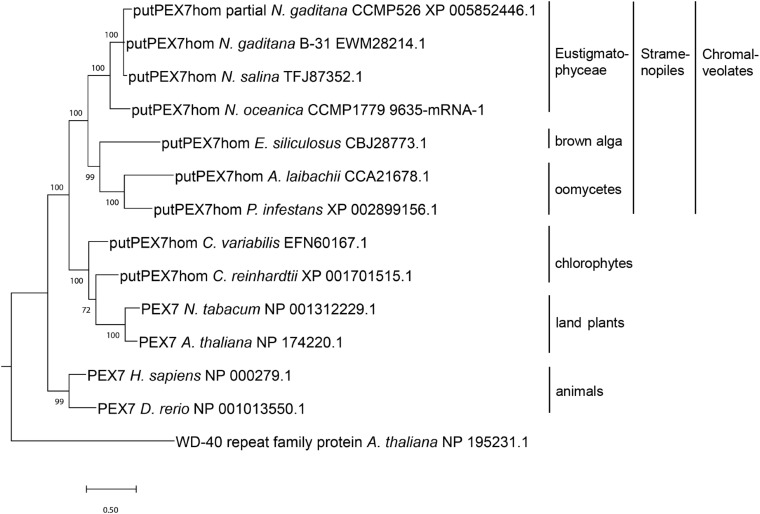
Phylogenetic analysis of PEX7 homologs detected in stramenopiles and related organisms. Sequences were aligned by MUSCLE (Edgar, 2004) and the phylogenetic tree was constructed using the Bayesian inference method (Ronquist et al., 2012). The branch support values were calculated as Bayesian posterior probabilities and are shown next to the branches. The platform Phylogeny.fr (Dereeper et al., 2008) was used for phylogenetic analysis, and the tree was visualized by MEGA X (Kumar et al., 2018).

Phylogenetic analysis of putative peroxisomal proteins was conducted on the platform Phylogeny.fr (Dereeper et al., 2008). Multiple sequence alignments were created by the MUSCLE algorithm (v3.8.31, Edgar, 2004). Phylogenetic trees were reconstructed using the Bayesian inference method, which is based on the Poisson model implemented in the MrBayes program (v3.2.6, Ronquist et al., 2012). The rate variation across sites was fixed to “invgamma”, and the number of substitution types was set to 6. Four Markov Chain Monte Carlo chains were run for 10,000 generations (sampling every 10 generations) with the first 250 sampled trees discarded as “burn-in”. Last, a “50% majority rule consensus tree” was constructed. The percentage of posterior probabilities of replicate trees, in which the associated taxa clustered together in the support test, is shown next to the branches (see Figure 1 and Supplementary Figures 5, 9). Graphical representation and formatting of the phylogenetic trees were performed with MEGA X (Kumar et al., 2018).

### Molecular Cloning

Genomic DNA was isolated from *N. gaditana* strain CCMP526 as described (Vieler et al., 2012) with the DNAeasy^®^ Mini Kit (Qiagen, Germany). The N-terminal domains of NgMLS2 (B-31, EWM30341.1, 44 aa including RIx_5_HL), PKT (EWM24705.1, 24 aa including RLx_5_HL), and histidine triad family protein 1 (HIT1, EWM29206.1, 68 aa including RLx_5_HL) were amplified with primers including specific restriction sites (Supplementary Table 3) and inserted directly into the pCAT vector upstream of enhanced yellow fluorescent protein (EYFP) (Ma et al., 2006). Amplified DNA was sequenced to verify the identity. Plasmid DNA for *in vivo* subcellular targeting analyses was extracted with the GeneJET Plasmid Miniprep Kit (Thermo Fisher Scientific^TM^, United States).

For subcellular localization studies in *Nannochloropsis*, the above-mentioned N-terminal domains of NgMLS2 and NgHIT1 and the full-length CDS of AtpMDH1 (At2g22780) were re-amplified from available pCAT vectors and ABRC clones, respectively (Supplementary Table 3), and inserted upstream of the Venus fluorophore in the *N. oceanica* transformation vector pNoc ox Venus (Zienkiewicz et al., 2017). To create a peroxisomal marker for *N. oceanica*, the original pNoc ox Venus vector was modified by exchanging the hygromycin resistance gene and the nopaline synthase terminator against the blasticidin resistance gene and a cauliflower mosaic virus (CaMV) 35S terminator. The latter elements were reamplified from an episomal CRISPR/Cas9 vector (Addgene accession number: #101009, Poliner et al., 2018; Supplementary Table 3) and subcloned by ApaI and NotI. The *Venus* reporter gene was replaced by the blue fluorescent reporter gene *mCerulean* extended by a C-terminal PTS1 (Falter et al., 2019) using the given primer pairs (Supplementary Table 3) and the restriction enzymes SgsI and SacI.

For *PEX7* expression analyses, RNA was isolated from *N. gaditana* CCMP526 as described (Vieler et al., 2012) using Trizol^TM^ (Invitrogen^TM^, United States) and the RNeasy^®^ Mini Kit with DNase digest (Qiagen, Germany). Total single strand cDNA was generated using the RevertAid First Strand cDNA Synthesis Kit and an oligo-dT primer (Thermo Scientific^TM^, United States), followed by PCR amplification of *PEX7* with specific primers (B-31, EWM28214.1; CCMP526, NGA_0680400; Supplementary Table 3). The specific 460-bp amplicon obtained with the primer pair of fw2 and rv1 was sequenced, which confirmed the cDNA identity.

### *In vivo* Subcellular Targeting Analyses

Onion (*Allium cepa* L.) epidermal cells were transformed biolistically as described (Falter et al., 2019), followed by microscopic analyses 1–7 days post transformation (dpt). *Arabidopsis* protoplasts were transiently transformed as described (Yoo et al., 2007) and analyzed 1–2 dpt. For *N. oceanica* CCMP1779 transformation, the wild-type cells were grown under standard growth conditions in f/2 medium (22°C, 75 μmol photons m^–2^ s^–1^, 16/8 h light/dark) to mid-exponential growth phase and transformed by electroporation with 3 μg of vector (linearized by AhdI), and 30 μg of salmon sperm DNA (Invitrogen), as described (Vieler et al., 2012; de Grahl et al., 2020). Single colonies were grown on selective plates containing 50 μg/ml hygromycin, transferred to 96-well plates containing 200 μl of f/2 medium with hygromycin and were incubated for 7–10 days under standard growth conditions (de Grahl et al., 2020) for subcellular analysis by confocal microscopy.

### Confocal and Transmission Electron Microscopy

For confocal microscopy, a Leica DMi8 inverted microscope was used coupled to a confocal spinning disc unit CSU X1 (Yokogawa Electric Corporation; Musashino, Japan). The system was equipped with a 445-nm laser for excitation of CFP and monomeric Cerulean and a 515-nm laser for excitation of EYFP and Venus with the corresponding emission filters (ET480/40m and ET535/30m, respectively). Image acquisition was performed using the VisiView software (Visitron Systems, Puchheim, Germany). Confocal images were captured as single planes with a QImaging OptiMOS sCMOS camera system.

For transmission electron microscopy (TEM), stable *N. oceanica* CCMP1779 transformants expressing *NgMLS2-Venus* were harvested in mid-exponential growth phase by centrifugation and were fixed with a mixture of 5 ml osmium tetroxide (1% in 0.1 M cacodylate buffer, pH 7.0), 2 ml sucrose [20 mM in 0.25× salinity artificial sea water (ASW)], 0.8 ml paraformaldehyde (16%) and 0.02 ml glutaraldehyde by incubation on ice for 1.5 h (Karlson et al., 1996). After four times washing with 0.25× salinity ASW, the cells were imbedded in 2% agarose. After dehydration with a graded ethanol series and LR-white-infiltration, ultrathin sections were obtained with an ultramicrotome (Ultracut E, Leica-Reichert-Jung, Nußloch, Germany). For immunogold labeling, a section containing the embedded algae was incubated with MSB (100 mM PIPES, 10 mM EDTA, 5 mM magnesium sulfate, pH 6.8) for 30 min and blocked with 3% BSA and 0.2% BSA-C in MSB for 30 min. After incubation with the primary anti-GFP antibody from rabbit (dilution 1:200; Abcam, Cambridge, United Kingdom), the section was washed five times with 1% BSA and 0.07% BSA-C in MSB and incubated with the secondary antibody [anti-rabbit IgG-Gold (10 nm), 1:50 dilution; Sigma Life Science, Taufkirchen, Germany] for 1 h and washed again. After a treatment with 1% glutaraldehyde and washing with water (three times), sections were incubated with 2% uranyl acetate for 10 s, washed with water once again and incubated with 0.2% lead citrate for 15 s. Sections were viewed with a LEO 906 E TEM (LEO, Oberkochen, Germany) equipped with the MultiScan CCD Camera (Model 794) of Gatan (Munich, Germany) using the Digital Micrograph software version 2.0.2. from Gatan to acquire, visualize, analyze, and process the image data.

## Results

### Prediction and Expression Analysis of a PEX7 Ortholog of *N. gaditana*

**FIGURE 2:**
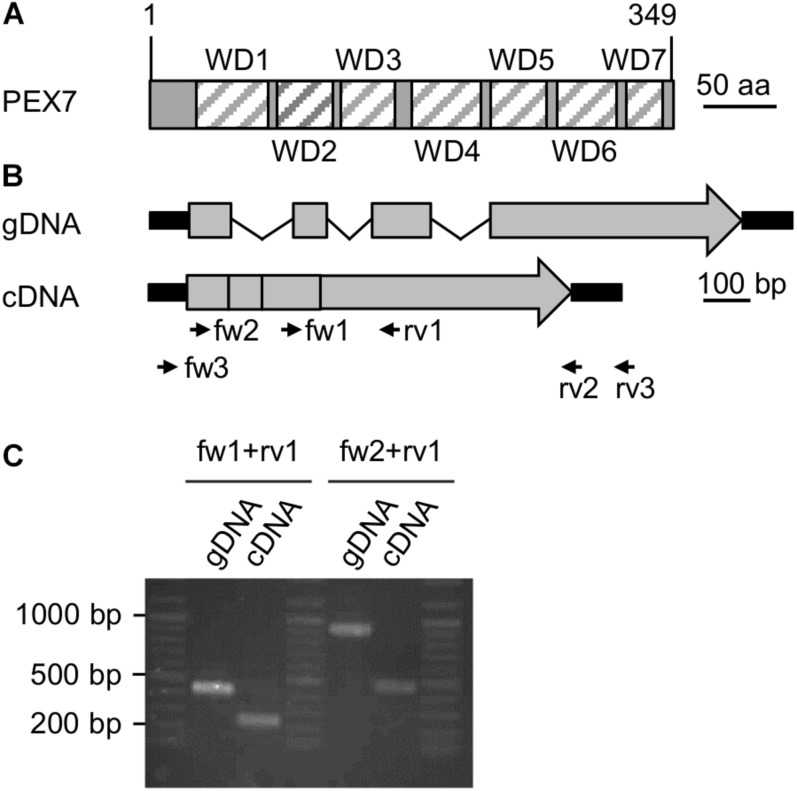
Expression analysis of a putative *PEX7* ortholog from *Nannochloropsis*. **(A)** Domain structure of the predicted PEX7 protein from *N. gaditana* with seven predicted WD40 domains (striped boxes, WD1-7). **(B)** Predicted gene and cDNA structure of *PEX7* from *N. gaditana*. Exons (gray boxes), introns (thin black lines) and UTRs (thick black lines) are indicated. The amplicon sizes were calculated for the primers fw1 + rv1 (gDNA: 420 bp; cDNA 240 bp) and for fw2 + rv1 (gDNA: 900 bp; cDNA: 460 bp). **(C)** PCR product size determination for gDNA and cDNA isolated from *N. gaditana*. The apparent sizes were consistent with the calculated sizes. Sequence analysis of the 460-bp amplicon obtained with fw2 and rv1 from cDNA confirmed its identity with the predicted cDNA and, hence, expression of the *PEX7* gene in *N. gaditana*.

In stramenopile algae, experimental studies investigating peroxisome biogenesis and functions are presently restricted to the diatom *Phaeodactylum*, which was shown to lack the PTS2 import pathway (Gonzalez et al., 2011). To investigate whether the absence of the PTS2 pathway is a general feature of stramenopiles or specific to one or several subgroups, we chose the family of *Eustigmatophyceae* and *Nannochloropsis* as a representative genus (Supplementary Figure 1). To identify PEX proteins encoded in the *N. gaditana* genome, those of *Arabidopsis thaliana* (Supplementary Table 1) were used as queries for homology searches. Orthologs of nearly all *A. thaliana* PEX proteins were identified in *N. gaditana* (Supplementary Table 1), suggesting that the fundamental mechanisms of peroxisome biogenesis and proliferation are largely conserved in *Eustigmatophyceae*. The detection of *Nannochloropsis* homologs for the PTS2 protein receptor, PEX7, as well as the docking complex proteins, PEX13 and PEX14, supported the existence of the import pathway for PTS2-carrying matrix proteins in this microalga. To investigate whether the identified *Nannochloropsis* protein was a true PEX7 ortholog, we performed phylogenetic analysis of this protein in comparison to other PEX7 orthologs. Indeed, the putative *Nannochloropsis* PEX7 homologs shared a common ancestor with PEX7 homologs of other stramenopiles, such as brown algae (*Ectocarpus*), *Pelagophyceae* (e.g., *Aureococcus*) and oomycetes (e.g., *Phytophthora*, Figure 1). The tree topology also showed that PEX7 of stramenopiles is more closely related to PEX7 orthologs from green algae and land plants than to animal PEX7. Similar to *Arabidopsis*, *Nannochloropsis* PEX7 contains seven predicted WD40 repeats (Figure 2A).

We next investigated experimentally whether the *PEX7* gene was indeed expressed and involved in peroxisome biogenesis or represented an untranscribed pseudogene without protein function *in vivo*. Total RNA was isolated from *N. gaditana* strain CCMP526 grown to logarithmic growth phase and converted to single strand cDNA using an oligo-dT primer for subsequent PCR amplification by three different *PEX7* primer pairs (Supplementary Table 3). While the full-length cDNA or CDS was not obtained, two different smaller fragments could indeed be amplified (Figure 2C). One primer pair binding to exons 3 and 4 amplified a 240-bp fragment from cDNA that clearly differed in size from the longer 420-bp fragment from genomic DNA (Figure 2B). The largest *PEX7* CDS fragment of 460 bp spanned from the beginning of exon 1 to the middle of the 4th and last exon. Sequence analysis confirmed its identity with *PEX7* and the lack of introns. The data demonstrated that *PEX7* was indeed expressed and indicated that the PTS2 protein import pathway was functional in *N. gaditana.*

### Prediction of PTS2 Cargo Proteins in *N. gaditana*

We next predicted the presence of putative PTS2 proteins by *in silico* analysis of the available genomes of *N. gaditana* strains and their deduced proteomes. We experienced that the protein predictions of *N. gaditana* strain B-31 were more reliable compared to those of *N. gaditana* CCMP526 and focused on the former. In brief, PTS2 containing proteins were identified in *N. gaditana* B-31 by a direct PTS2 search using a relatively stringent PTS2 motif, [RK][LVI]x_5_[HQ][LAF] (Kunze et al., 2011). More than 50 protein sequences were detected, 13 of which contained the PTS2 motif in the typical N-terminal 40-amino acid residue domain (Table 1). The putative PTS2 proteins included few homologs of *Arabidopsis* PTS2 proteins, including one malate synthase (referred to as NgMLS2; EWM30341.1) of the glyoxylate cycle and one (peroxisomal) 3-ketoacyl-CoA thiolase (NgPKT, EWM24705.1) involved in fatty acid β-oxidation. A third protein was homologous to the bifunctional *A. thaliana* transthyretin-like protein, also referred to as allantoin synthase (ALNS; Reumann et al., 2007; Lamberto et al., 2010). The C-terminal domain acts as a 5-hydroxyisourate hydrolase (HIUase, Supplementary Figure 2). Another *Nannochloropsis* protein was annotated as “protein kinase c binding protein” (EWM29206.1) and homologous to the *Arabidopsis* HIT3, which is a PTS2 protein of yet unknown physiological function identified in the peroxisomal proteome of *Arabidopsis* leaf peroxisomes (Reumann et al., 2009). The *N. gaditana* protein is referred to as NgHIT1 (Table 1). Hence, out of eleven known PTS2 protein families in *Arabidopsis* (Supplementary Table 2), four had predicted PTS2 homologs also in *Nannochloropsis* (Table 1 and Supplementary Table 2), further supporting the presence of PTS2 cargo and the existence of the PTS2 pathway in *Nannochloropsis*.

Interestingly, most of the known *Arabidopsis* PTS2 protein families had predicted PTS1- rather than PTS2-carrying homologs in *Nannochloropsis* (Supplementary Table 2), namely two acyl-CoA oxidases (both ARL>), one aspartate aminotransferase (AHL>), one citrate synthase (ARL>), one long-chain acyl-CoA synthetase (ARL>), and the N-terminal domain of the bifunctional *A. thaliana* transthyretin-like protein, which decarboxylates 2-oxo-4-hydroxy-4-carboxy-5-ureidoimidazoline (OHCU, SRL>, Supplementary Figure 2) as part of urate catabolism. Hence, key metabolic functions of *Arabidopsis* PTS2 proteins (glyoxylate cycle, fatty acid β-oxidation) appeared as being conserved in *Nannochloropsis* peroxisomes but seemed to be carried out by PTS1 homologs instead. Four other *Arabidopsis* proteins/families had apparent non-peroxisomal homologs in *N. gaditana*, including (i) malate dehydrogenase (MDH, but SHL> in *N. oceanica* CCMP1779), (ii) pseudouridine monophosphate glycosylase/indigoidine synthase A (PUMY/IndA), which forms a bifunctional fusion protein together with pseudouridine kinase/6-phosphofructokinase (PUKI/PfkB, Supplementary Figure 3), both of which are involved in (peroxisomal) pseudouridine catabolism in *Arabidopsis* (Chen and Witte, 2020), (iii) 1,4-dihydroxy-2-naphthoyl-CoA synthase (DHNS) involved in phylloquinone biosynthesis (Babujee et al., 2010), and (iv) one small heat-shock protein with an alpha-crystallin domain (Ma et al., 2006; Supplementary Table 2).

The other *N. gaditana* proteins with predicted PTS2 (Table 1) were not homologous to known *Arabidopsis* PTS2 proteins and indicated novel functions of *Nannochloropsis* peroxisomes. They included, for instance, an α/β-hydrolase domain-containing protein, also annotated as embryogenesis-associated protein EMB8 (EWM21473.1, RLx_5_QL), which atypically also contained a predicted PTS1 (SRL>).

Similar PTS2 protein predictions were carried out for the second *N. gaditana* strain CCMP526 and for *N. oceanica* CCMP1779, both of which further confirmed the PTS2 protein predictions. Atypically and interestingly, one predicted PTS2 protein of *N. gaditana* B-31 (EWM27137.1, hypothetical protein, RLx_5_HL) had a PTS1-containing homolog in *N. oceanica* CCMP1779 (protein ID: 564055, SRL>), strengthening the predicted peroxisome targeting of both orthologs. Predicted peroxisome targeting of these homologs in stramenopiles or other taxa indicated novel peroxisomal proteins and functions.

We validated the PTS2 protein predictions of *N. gaditana* by different complementary computational analyses, for instance, PTS2 conservation analysis in other photosynthetic stramenopiles, such as *Nannochloropsis* (*N. oceanica*, *N. salina*), *Ectocarpus*, *Aureococcus* (*Pelagophyceae*) and in heterotrophic stramenopiles, such as oomycetes (Supplementary Figure 1). The PTS2 of some proteins was well conserved (Supplementary Figure 4). For NgPKT, for instance, predicted PTS2 nonapeptides were detected in nearly all orthologs of *Nannochloropsis*, *Ectocarpus*, oomycetes and even in two early branching species (*Hondaea fermentalgiana*, slime net, and *Cafeteria roenbergensis*, Supplementary Figure 4A). In further support of true functional PTS2, all NgPKT orthologs contained hydrophobic residues at position 5 of their nonapeptides (Ile or Leu, Supplementary Figure 4A). In the four diatoms, the orthologs were N-terminally shortened by approximately 20 aa and possessed a PTS1 instead (SRL>, SSL>, and SAL>, data not shown). Hence, PTS2 conservation in stramenopiles outside of *Bacillariophyceae* (diatoms) together with PTS1 presence in diatoms in many stramenopile orthologs strongly supported the correct PTS2 prediction in NgPKT. By contrast, due to a lack of NgMLS2 orthologs in stramenopiles, PTS2 conservation analyses did not provide further support for correct PTS2 prediction in NgMLS2 (data not shown). Other predicted *Nannochloropsis* PTS2 proteins either lacked obvious orthologs in other stramenopiles or the PTS2 of the *Nannochloropsis* protein was not conserved (data not shown).

Because proteins rarely contain simultaneously two different N-terminal targeting signals to both peroxisomes (PTS2) and a second cell organelle (ER, mitochondria, plastids), we also investigated whether any PTS2 proteins were simultaneously predicted to be targeted to any non-peroxisomal cell organelle in stramenopiles, which would weaken our PTS2 prediction (e.g., HECTAR^1^; Gschloessl et al., 2008). Indeed, many putative PTS2 proteins were not predicted to additionally possess an N-terminal targeting signal for mitochondria, complex plastids or the ER (data not shown) except for NgHIT1 (see below).

In spermatophytes, the PTS2 is cleaved off in the peroxisomal matrix by the protease DEG15 (SKL>, Helm et al., 2007; Schuhmann et al., 2008; Dolze et al., 2013). A putative but weakly conserved homolog of DEG15 was indeed detected in *Nannochloropsis* (EWM21659). It could be aligned with AtDEG15 and putative stramenopile orthologs and clustered reasonably upon phylogenetic analysis (Supplementary Figures 5A,B). Peroxisome targeting and proteolytic activity of the *Nannochloropsis* homolog are supported by the presence of a predicted PTS1 (SLL>) and conservation of the catalytic triad (His-Asp-Ser, data not shown), respectively. The cleavage site of PTS2 proteins is characterized by a conserved Cys-containing motif closely downstream of the PTS2 nonapeptide (Kato et al., 1995, 1996). When searching for this feature, both NgPKT and NgHIT1 (but not NgMLS2) indeed contained such Cys (Supplementary Figure 6). Taken together, all these features of the predicted PTS2 cargo proteins and of the DEG15 peptidase further supported the existence of a functional PTS2 protein import pathway in *Nannochloropsis*.

### *In vivo* Subcellular Targeting Analyses of Predicted PTS2 Proteins in Two Plant Expression Systems

For experimental validations of the predictions, we selected three putative PTS2 proteins (Table 1). To enhance surface exposure of the PTS2 peptide in the fluorescent protein fusion and to avoid oligomerization with endogenous isoforms and co-import by piggy-back mechanism, we restricted the gene fusion to the N-terminal exon (24–68 amino acid residues, Supplementary Figure 6) encoding the predicted PTS2. This domain was amplified from genomic DNA of *N. gaditana* CCMP526 and cloned into the transient vector, pJET. Sequence analysis verified cloning of the correct exons. The PTS2 domains were subcloned into the plant expression vector pCAT for expression from the strong CaMV 35S promoter and to create C-terminal fusion proteins with EYFP.

**FIGURE 3:**
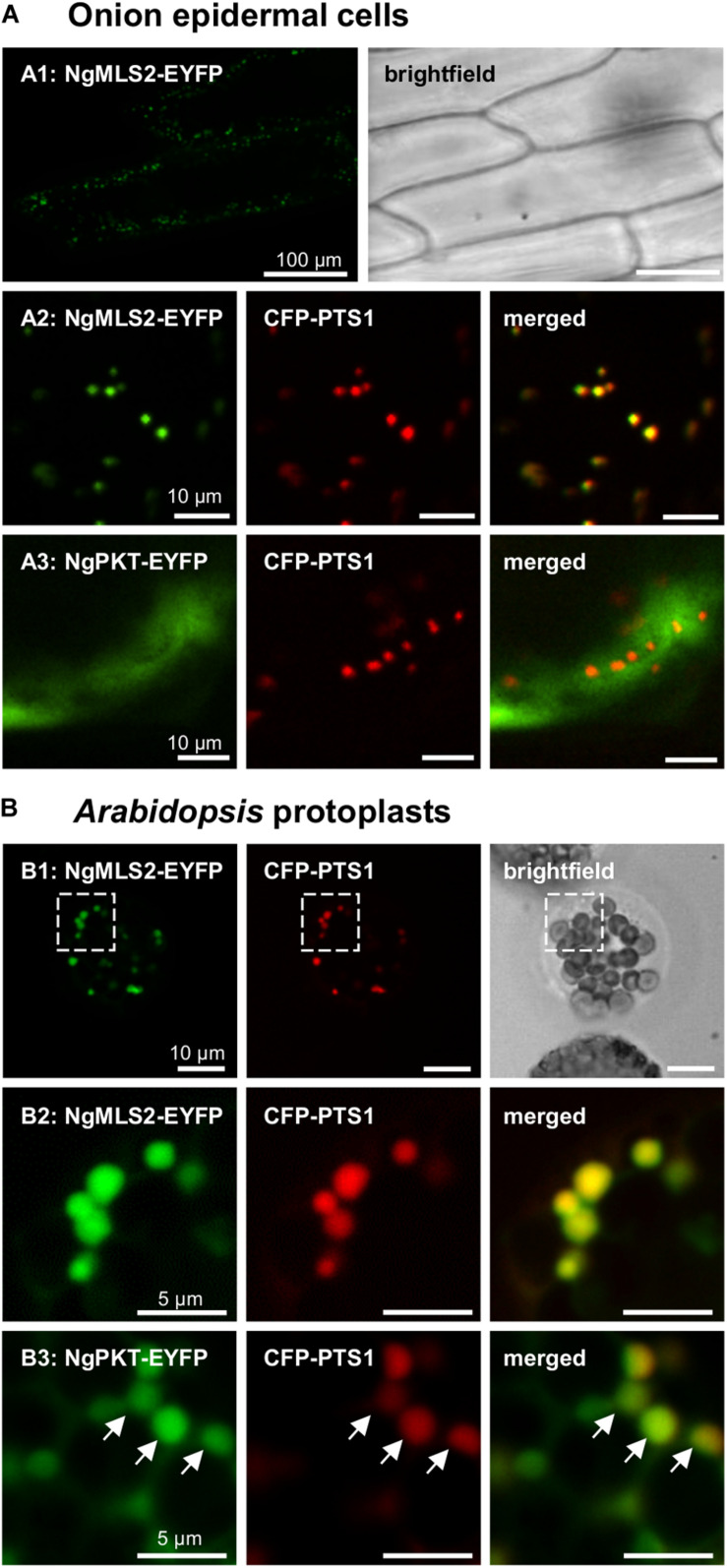
*In vivo* subcellular targeting analyses of two putative PTS2 proteins from *N. gaditana* in plant cells. **(A)** In onion epidermal cells, MLS2-EYFP co-localized with the peroxisome marker, CFP-PTS1 (already 1 dpt). However, NgPKT remained cytosolic, even after long-term expression of 7 dpt. The first exons of the putative PTS2 proteins from *N. gaditana* were fused C-terminally with EYFP and transiently co-expressed with CFP-PTS1 in onion epidermal cells. **(B)** In transiently transformed *Arabidopsis* protoplasts, NgMLS2-EYFP also co-localized with the peroxisomal marker (2 dpt). The boxed region shown in B1 is enlarged in B2. NgPKT-EYFP remained largely cytosolic but showed partial co-localization with the peroxisome marker 2 dpt. The arrows point out peroxisome targeting of NgPKT-EYFP.

In the transient monocotyledon expression system of onion epidermal cells, the predicted PTS2 domain of NgMLS2 (RIx_5_HL) directed EYFP to small punctate structures that were identical with peroxisomes, as demonstrated by co-expression with the peroxisomal marker, cyan fluorescent protein (CFP-SKL, Figures 3A1, A2). EYFP alone served as negative control (data not shown). Also in the dicotyledon expression system of *Arabidopsis* protoplasts, the predicted PTS2 domain of NgMLS2 was sufficient to direct EYFP to peroxisomes (Figures 3B1, B2). Despite its canonical PTS2, however, the second PTS2 protein, NgPKT-EYFP (RLx_5_HL), remained cytosolic in onion epidermal cells even after extended expression times of 7 days (Figure 3A3). In *Arabidopsis* protoplasts, where weak peroxisome targeting can often be detected with higher sensitivity (Kataya and Reumann, 2010), the same fusion protein was targeted to peroxisomes in several cells, consistent with its predicted PTS2 (Figure 3B3). We concluded that both NgMLS2 and NgPKT were indeed peroxisomal matrix proteins of *N. gaditana* and were targeted to the organelle by the PTS2 pathway. While NgMLS2 was efficiently imported into peroxisomes in both plant expression systems, NgPKT was weakly imported into plant peroxisomes for yet unknown reasons.

**FIGURE 4:**
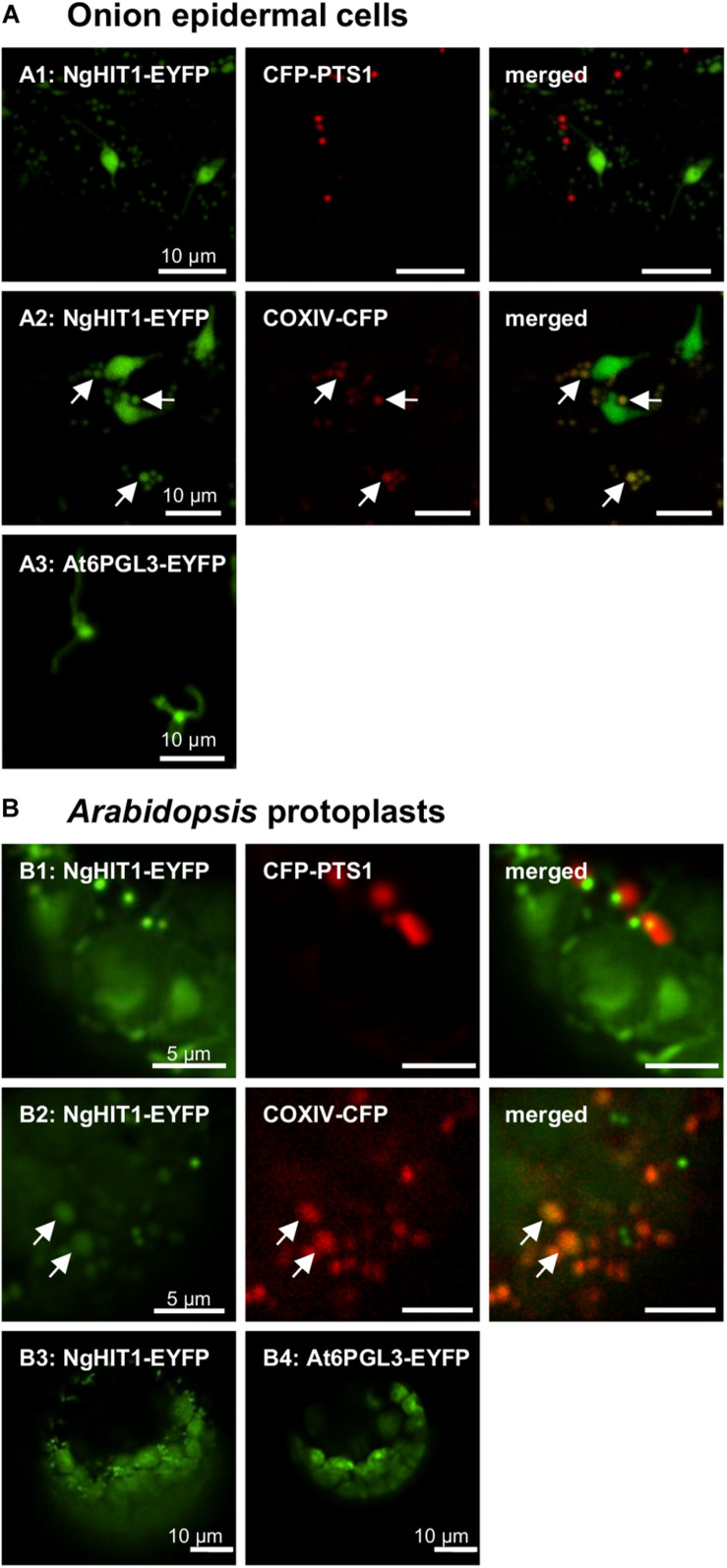
*In vivo* subcellular targeting analyses of a HIT homolog from *N. gaditana* in plant cells. **(A)** In onion epidermal cells, the putative PTS2 protein NgHIT1 was targeted to punctate structures of different sizes, some of which were larger than and not identical with peroxisomes labeled by CFP-PTS1 (A1). Yellow fluorescent organelle extensions were reminiscent of leucoplast stromuli **(A1)**. Co-expression with a mitochondrial marker, COXIV-CFP, showed that NgHIT1 was partly targeted to mitochondria **(A2)**. A known plastidic protein from *Arabidopsis*, At6PGL3-EYFP, labeled leucoplasts and their stromuli **(A3**; Reumann et al., 2007), showing that NgHIT1-EYFP was dually targeted to both plastids and mitochondria. **(B)** Likewise, in *Arabidopsis* protoplasts, NgHIT1 was targeted to small organelle-like structures, not identical with but frequently found in close proximity to peroxisomes **(B1)**. Co-expression with the mitochondrial marker (COXIV-CFP) identified the organelles as mitochondria **(B2)**. As in onion epidermal cells, NgHIT1-EYFP was also targeted to plastids (chloroplasts, **B3)**, as was At6PGL3-EYFP (Reumann et al., 2007). Images were taken 2 dpt. The arrows point out mitochondrial targeting of NgHIT1-EYFP.

The third representative protein, NgHIT1, showed a different subcellular targeting contrary to the PTS2 predictions. In onion epidermal cells, NgHIT1-EYFP labeled punctate structures that were not identical with and larger than peroxisomes. These structures had terminal extensions that were reminiscent of plastidic stromuli (Figures 4A1, A2). The fluorescent pattern of NgHIT1-EYFP was similar to that of *Arabidopsis* 6-phosphogluconolactonase 3 (AtPGL3-EYFP), which localizes to leucoplasts in onion epidermal cells and labels their stromuli extensions, as reported earlier (Figure 4A3; Reumann et al., 2004, 2007; Holscher et al., 2014). Consistent with the prediction of a mitochondrial presequence by HECTAR (score: 0.8, Gschloessl et al., 2008), NgHIT1-EYFP was additionally directed to mitochondria labeled with COXIV-CFP (Figure 4A2). Similar results of dual protein targeting to both mitochondria (Figures 4B1, B2) and plastids (Figure 4B3) were obtained in *Arabidopsis* protoplasts, where plastid targeting was even better detectable due to the larger plastids and their chlorophyll autofluorescence. In conclusion, two (out of three) predicted and experimentally investigated *Nannochloropsis* PTS2 proteins were indeed imported into peroxisomes in *Arabidopsis* protoplasts, confirming the existence of PTS2 cargo proteins in *N. gaditana*.

### Peroxisome Targeting Analyses in *Nannochloropsis*

**FIGURE 5:**
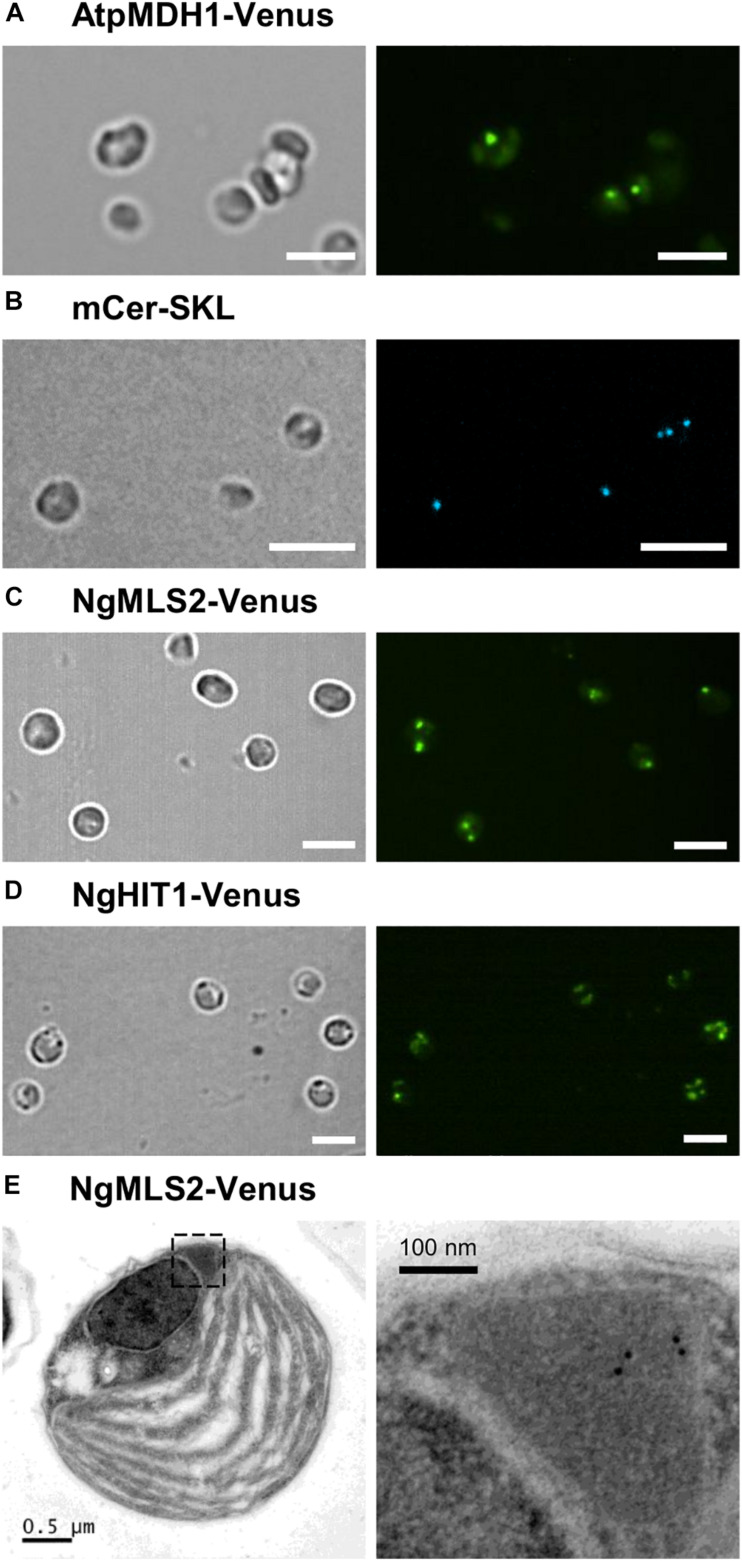
Peroxisome targeting analysis of representative *Arabidopsis* and *Nannochloropsis* PTS2 proteins in *N. oceanica*. **(A)** As positive control, the PTS2 protein, pMDH1 from *A. thaliana* (AtpMDH1), was C-terminally fused to Venus in the expression vector pNoc ox Venus. Wild-type *N. oceanica* was transformed by electroporation, and stable transformants were generated. Venus fluorescence was visible as small puncta indicative of AtpMDH1 targeting to peroxisomes. **(B)** To label peroxisomes, *N. oceanica* wt cells were transformed with an expression vector encoding blue fluorescent monomeric Cerulean extended by SKL> (mCer-SKL). **(C)** The first exon of NgMLS2 containing the predicted PTS2 was chosen representatively and fused similarly with Venus. In the corresponding *N. oceanica* transformants, Venus fluorescence was visible in a single small punctate structure, consistent with Venus targeting to *Nannochloropsis* peroxisomes. In some transformants, two or more punctate fluorescent structures were visible. Scale bar 5 μm. **(D)** NgHIT1-Venus was also targeted to small punctate structures, but additionally to larger round structures reminiscent of chloroplasts. Scale bars 5 μm in **(A–D)**. **(E)** For validation of NgMLS2-Venus targeting to peroxisomes, TEM was carried out combined with immunogold-labeling using a primary antibody against GFP. Gold particles (10 nm) were specifically detected in peroxisomes. Scale bars as indicated.

In order to investigate peroxisome targeting of predicted PTS2 proteins also in the homologous system of *Nannochloropsis*, selected PTS2 domains were subcloned behind the endogenous strong and constitutive promoter of elongation factor and in front of the *Venus* gene in an appropriate *N. oceanica* expression vector (pNoc ox Venus, Supplementary Table 3; Zienkiewicz et al., 2017; de Grahl et al., 2020). As a well-known heterologous PTS2 cargo protein, we used *Arabidopsis* peroxisomal malate dehydrogenase 1 (pMDH1, At2g22780). *N. oceanica* CCMP1779 was transformed by electroporation, and transformants were selected by hygromycin resistance. Single transformants were grown under standard growth conditions in liquid cultures in 96-well plates and were analyzed after 6–8 days by spinning disk confocal microscopy. Not only AtpMDH1 but also NgMLS2 directed Venus to small punctae (approximately 1–2 per cell) of high fluorescence, consistent with peroxisome targeting (Figures 5A,C). Punctae of similar size and shape were detected for a peroxisomal marker, i.e., monomeric blue fluorescent Cerulean, extended C-terminally by a PTS1 (mCer-SKL, Figure 5B). The small punctae labeled by NgHIT1-Venus, however, were more numerous per cell (Figure 5D). Hence, the punctae labeled by AtMDH1 and NgMLS2 provided additional indirect evidence for maintenance of the PTS2 protein import pathway in *Nannochloropsis*.

To further validate PTS2 cargo targeting to peroxisomes by the PTS2 import pathway in *N. oceanica*, we investigated peroxisome targeting of NgMLS2-Venus by TEM, using primary antibodies against GFP and gold-labeled secondary antibodies. Peroxisomes were well distinguishable from chloroplasts, mitochondria, lipid bodies, and vacuoles by their size, the lack of internal membranes and by their protein-rich matrix. Approximately 60% of the visible algal peroxisomes were labeled at least by one gold particle per peroxisome, and approximately half of these contained several gold particles (Figure 5E). In contrast, no gold-labeled peroxisomes could be identified in the untransformed wild-type strain. Although a minor background of gold labeling was observed, no EYFP targeting to any other organelles like mitochondria or chloroplasts was visible. Thus, the TEM results provided complementary support for the identification of NgMLS2 as a true PTS2 cargo and functionality of the PTS2 protein import pathway in *Nannochloropsis*.

**FIGURE 6:**
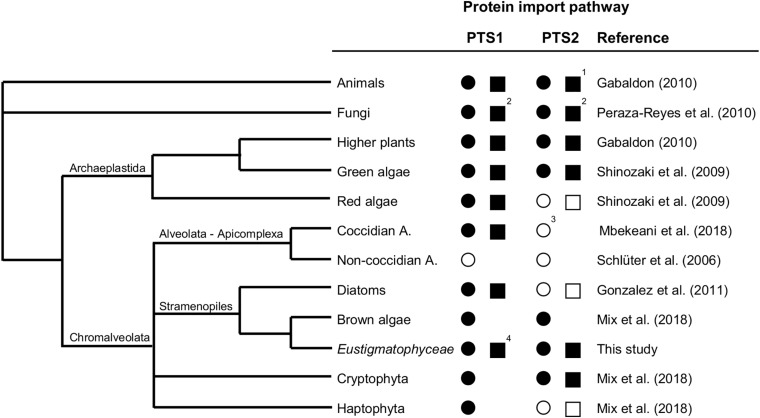
Existence and secondary loss of the peroxisomal targeting pathways for PTS1 and PTS2 proteins in different taxa. The predicted existence of the PTS1 and PTS2 protein import pathways are denoted as a filled circle (present in all representative organisms sequenced to date) or empty circle (absent in all representative organisms sequenced to date). Experimentally verified existence of the PTS1 and PTS2 protein import pathways are denoted as a filled square (presence shown at least for one representative organism) or empty square (absence demonstrated for one representative organism). ^1^Among animals, the PTS2 pathway has experimentally been shown to be absent in *C. elegans* and *D. melanogaster*. ^2^Among fungi, peroxisomes are missing in the rumen fungi, Neocallimasticalles and Microsporidia. ^3^In coccidian apicomplexa (e.g., *Toxoplasma*), which possess peroxisomes contrary to non-coccidian apicomplexa (e.g., *Plasmodium*), the absence of the PTS2 pathway is indicated by the lack of PEX7, PEX13, and PEX14, even though PEX5 possesses the typical PEX7 binding domain. ^4^Full functionality of the PTS1 import pathway has been demonstrated (Kechasov, 2017; Kechasov et al., in preparation). A., Apicomplexa.

Taken together, by (i) verifying the expression of the cytosolic PEX7 receptor, (ii) predicting more than a dozen of PTS2 cargo proteins, and (iii) experimentally validating peroxisome targeting of representative PTS2 candidates in plants and *Nannochloropsis*, we concluded that the PTS2 protein import pathway of peroxisomes has been maintained in full functionality in the stramenopile alga *Nannochloropsis*, which contrasts peroxisome biogenesis in the sister group of diatoms (Figure 6).

## Discussion

Land plants have an impressive and the largest number of PTS2 proteins that are directed to the peroxisomal matrix by the PTS2 import pathway, as exemplified by 20 PTS2 proteins in *A. thaliana* (Supplementary Table 2). They include very important enzymes of fatty acid β-oxidation (e.g., three acyl-CoA oxidases, three thiolases and long-chain acyl CoA synthetase 6) and of the glyoxylate cycle (three citrate synthases, two MDHs). Additional PTS2 proteins of *Arabidopsis* participate, for instance, in purine breakdown (transthyretin/ALNS, Reumann et al., 2007; Lamberto et al., 2010), pseudouridine catabolism (pseudouridine monophosphate glycosylase, Reumann et al., 2009; Reumann, 2011; Chen and Witte, 2020), and phylloquinone biosynthesis (DHNS; Babujee et al., 2010; Reumann, 2013). Given the large number, diversity and importance of these metabolic pathways, knowledge of the existence of the PTS2 import pathway in *Nannochloropsis* is prerequisite not only to understand evolution, speciation, and peroxisome biogenesis in *Eustigmatophyceae*, but also to be able to comprehensively map and model all metabolic pathways and competencies of peroxisomes in this genus by computational predictions, followed by experimental validations.

PEX7 is the cytosolic receptor that binds the PTS2 of cargo proteins and shuttles them to the PEX13/14 docking complex in the peroxisomal membrane (Woodward and Bartel, 2005; Ramon and Bartel, 2010). In organisms that lack the PTS2 import pathway, the *PEX7* gene is generally absent (e.g., *C. elegans*, Motley et al., 2000; red algae, Shinozaki et al., 2009; *P. tricornutum*, Gonzalez et al., 2011). The only exception is *D. melanogaster* which possesses a functional full-length *PEX7* gene (Baron et al., 2016), but lacks PTS2 cargo proteins identifiable by known PTS2 peptides. Our detection of a predicted *PEX7* gene in *N. gaditana* B-31 is consistent with the report of a *PEX7* gene in *N. gaditana* strain CCMP526 (Lauersen et al., 2016) and in *N. oceanica* CCMP1779 (Mix et al., 2018). The domain structure of the *N. gaditana* PEX7 homolog shows seven typical WD-40 repeats of approximately 40 amino acid residues that often terminate with Trp-Asp (Neer et al., 1994). The phylogenetic relationship of NgPEX7 with PEX7 orthologs of brown algae (*E. siliculosus*) and oomycetes confirmed its true PEX7 orthology (Figure 1). The closer relationship of stramenopile PEX7 to orthologs from green algae and higher plants than to animal PEX7 indicates its inheritance from the engulfed red alga upon endosymbiosis rather than from the heterotrophic host genome. Contrary to *Phaeodactylum* and consistent with maintenance of the PTS2 import pathway in *Nannochloropsis*, the predicted orthologs of the PTS1 protein receptor, PEX5, from *N. gaditana* and *N. oceanica* indeed possess the conserved middle domain of approximately 30 amino acid residues that binds PEX7 in *A. thaliana* and *Homo sapiens* (Supplementary Figure 7; Dodt et al., 2001), further indicating maintenance of the PTS2 import pathway. We also found PEX13 and PEX14 orthologs in *N. gaditana* strain B-31, both of which are required for PTS2 and PTS1 matrix protein import (Cross et al., 2016).

Experimental evidence that the *PEX7* gene of *Nannochloropsis* was indeed expressed had not been reported but is crucial for PTS2 import pathway functionality. We could verify by RT-PCR that the region corresponding to the N-terminal half of PEX7 was indeed expressed in *N. gaditana* CCMP526 (Figure 2C), but all attempts to amplify the full-length *PEX7* CDS from mRNA failed for yet unknown and most likely technical reasons. Since an oligo-dT primer had been used for single-strand cDNA synthesis from mRNA, we consider it very likely that *PEX7* is indeed expressed in full length in *N. gaditana*. *PEX7* gene expression in *Nannochloropsis* is further supported by transcriptomics data of *N. gaditana* strain B-31 (i.e., Naga_100004g18 in table S17 of Corteggiani Carpinelli et al., 2014) and strain CCMP526 (i.e., Nga06804 in http://nannochloropsis.genomeprojectsolutions-databases.com/Expression_comp.xls for CCMP526, Radakovits et al., 2012). Furthermore, we identified a full-length *PEX7* mRNA in *N. oceanica* strain CCMP1779 in the JGI database (i.e., “583747” in https://mycocosm.jgi.doe.gov/cgi-bin/dispGeneModel?db=Nanoce1779_2&tid=583747, Vieler et al., 2012) that is highly similar to PEX7 of *N. gaditana* and covers the entire CDS (Supplementary Figure 8). Hence, *PEX7* is undoubtedly expressed in full-length in *N. oceanica*. Taken together, all these *PEX7* expression data of both species strengthen the indications that *Nannochloropsis* possesses a functional PTS2 import pathway.

The computational search for predicted PTS2 proteins in *N. gaditana* yielded, after filtering for the location of the PTS2 motif in the N-terminal 40-amino acid residue domain, 13 predicted PTS2 proteins. This filter was reasonable because, to the best of our knowledge, only two proteins are currently reported to carry the PTS2 at an internal position, namely ascomycete PEX8 and the plant transthyretin-like protein/ALNS involved in urate catabolism to allantoin (Reumann et al., 2007; Pessoa et al., 2010; Lamberto et al., 2010). Our evolutionary analyses revealed that the bifunctional plant enzyme arose in *Viridiplantae* shortly after divergence of rhodophytes in the last common ancestor of *chlorophyceae* and streptophytes by fusion of two single polypeptides, thereby placing the PTS2 internally (Supplementary Figure 2C). Stramenopiles evolved independently different improvements of protein structure and compartmentalization of purine catabolism. In diatoms (lacking the PTS2 pathway), both enzymes were fused but remained cytosolic. The extreme C-terminal four residues of HIUase (YRGS>), which are part of the active site, will not have allowed any PTS1 evolution. In *Nannochloropsis*, however, both enzymes stayed separated and were directed to peroxisomes (HIUase, RLx_5_HL; OHCU-DC, SRL>, Table 1 and Supplementary Figure 2D). Despite considerable sequence variation, the PTS2 of NgHIUase is very well conserved in diverse stramenopile orthologs, including a conserved hydrophobic residue at position 5 (Leu, Ile or Val, Supplementary Figure 4C). Hence, even though not investigated experimentally in this study, NgHIUase is most probably another correctly predicted PTS2 protein of *N. gaditana*.

Interestingly, many predicted PTS2 proteins of *Nanno*- *chloropsis* have in common that the PTS2 nonapeptide lies in the short first exon, while the functional protein domain generally starts with the 2nd exon. New PTS2 proteins may have been created by exon shuffling, placing the PTS2 containing first exon in front of novel genes to direct the gene products to peroxisomes. Exon shuffling can also result in alternative splicing of PTS2-encoding genes affecting subcellular localization of the proteins, as documented for *A. thaliana* (An et al., 2017).

The PTS2 motif [RK]-[LVI]-x_5_-[HQ][LAF] used for our *Nannochloropsis* genome screen was rather stringent (Kunze et al., 2011). More relaxed PTS2 motifs have been reported (Petriv et al., 2004; Reumann and Chowdhary, 2018). PTS2 conservation analysis of the newly detected *N. gaditana* PTS2 proteins in stramenopiles (Supplementary Figure 4) shows that most aligned nonapeptides are included in the motif, but notably not all. For instance, also Met and Val were found at position 2 (RMx_5_HL in *Ectocarpus* PKT; RVx_5_HL in *Phytophthora cactorum* HIUase) and at position 9 in several species (e.g., in RIx_5_HM in *Achlya* PKT, RLx_5_HV in *Saprolegnia* HIUase, Supplementary Figures 4A,C). Hence, at least these four nonapeptides are likely additional functional PTS2 nonapeptides in stramenopiles and shall be validated experimentally. Their application in future genome searches might allow the identification of additional PTS2 cargo proteins in *Nannochloropsis*.

Only few stramenopile microalgae can nowadays be transformed (Rathod et al., 2017). Moreover, *in vivo* subcellular targeting analyses of *N. oceanica* require 6–8 weeks from transformation to microscopy of liquid cultures (de Grahl and Rout, personal communication; de Grahl et al., 2020). The PTS1 and PTS2 motifs of both targeting pathways are well conserved in eukaryotes across kingdoms. Therefore, it is reasonable to investigate peroxisome targeting of microalgal proteins in transient expression systems of land plants. Indeed, two predicted PTS2 proteins from *N. gaditana* were correctly imported into plant peroxisomes (Figures 3A,B), confirming them as functional PTS2 nonapeptides and the proteins as correctly predicted PTS2 cargos. To validate these results in a homologous system, we expressed selected *Nannochloropsis* constructs also in *N. oceanica*. Not only *Arabidopsis* pMDH1 and the peroxisome marker, mCer-SKL, were targeted to small cell organelles, but also NgMLS2 (Figures 5A,B). The new blue fluorescent peroxisome marker, mCer-SKL, created in another expression vector containing a second resistance marker, shall allow *in vivo* co-localization studies in *N. oceanica* in future studies. TEM analysis identified the labeled structures as peroxisomes rather than mitochondria (Figure 5E). These results provided another independent line of evidence, leading altogether to the conclusion that the PTS2 import pathway remained fully active and functional in *Nannochloropsis*.

The enzyme malate synthase (MLS) is part of the peroxisomal glyoxylate cycle and very important for condensation of fatty acid-derived acetyl-CoA with glyoxylate to malate for carbohydrate synthesis (Kornberg and Krebs, 1957). Many organisms, such as plants, fungi, and animals (except for mammals) possess a single and PTS1-carrying MLS isoform. The same MLS isoform had previously been identified in representative genomes of chlorophytes and stramenopiles, but was absent in multicellular rhodophytes (i.e., *Chondrus crispus*) and haptophytes (i.e., *E. huxleyi*, Lauersen et al., 2016). While the authors detected only this single, PTS1-carrying MLS isoform in *N. gaditana* (EWM22958.1, SRL>, NgMLS1), we identified the second PTS2-carrying NgMLS2 in *N. gaditana* B-31 (NgMLS2, 52% sequence similarity with NgMLS1, Table 1). Due to a lack of orthologous isoforms in stramenopiles, PTS2 conservation analyses did not provide further support for correct PTS2 prediction in NgMLS2 (data not shown). Our experimental analyses demonstrated that NgMLS2 indeed possesses a functional PTS2 (RIx_5_HL) because the 1st exon of 68 aa in length was sufficient to direct EYFP to peroxisomes in two plant expression systems (Figures 3A,B). Also when expressed in *N. oceanica*, EYFP was detected in small fluorescent organelles that lacked internal membranes according to TEM analyses and were identified as peroxisomes rather than mitochondria (Figures 5C,E). Hence, NgMLS2 is a second MLS isoform of the peroxisomal glyoxylate cycle and a PTS2 cargo of the PTS2 import pathway in *Nannochloropsis*.

Interestingly, phylogenetic analysis showed that NgMLS2 and its few orthologs detected in specific oomycetes (e.g., *Saprolegnia diclina* VS20, *Phytophthora parasitica*), clusters together with the single (PTS1-containing) MLS isoform of animals in one clade (Supplementary Figure 9). Hence, the PTS2-carrying NgMLS2 is most likely of metazoan origin and stems from the heterotrophic host cell that engulfed the red alga approximately 1260 Mio years ago (Yoon et al., 2002). The 3rd detected MLS isoform (NgMLS3, EWM30541.1) is only distantly related to NgMLS1/2 and is most likely non-peroxisomal due to the lack of any predicted PTS1/2 (Supplementary Figure 9). By contrast, the PTS1-containing MLS1 isoform of *N. gaditana* is more closely related to those of the green lineage (e.g., chlorophytes, *Volvox carteri*; *A. thaliana*; *P. tricornutum*; and brown alga, *E. siliculosus*, Supplementary Figure 9). It will be interesting to learn why *Nannochloropsis* atypically maintained both the metazoan-like NgMLS2 and the plant-like NgMLS1 in peroxisomes and whether both enzymes specialized on slightly different functions.

3-Ketoacyl-CoA thiolase (also called thiolase) is a degradative key enzyme of fatty acid β-oxidation with broad chain-length specificity for its substrates. We found a single ortholog in *N. gaditana*, while the second *N. gaditana* thiolase (EWM30137.1) is a biosynthetic enzyme and orthologous to *Arabidopsis* acetyl-coenzyme A acetyltransferase, which converts two acetyl-CoA to acetoacetyl-CoA in the mevalonate pathway. The PTS2 of NgPKT was well conserved among stramenopile orthologs, which strongly supported the correct PTS2 prediction in NgPKT. However, when investigating peroxisome targeting of NgPKT experimentally *in vivo*, the N-terminal 22-aa peptide of this protein did not detectably direct EYFP to peroxisomes in onion epidermal cells, even after long-term expression times (Figure 3A). In *Arabidopsis* protoplasts, however, peroxisome targeting was better and clearly detectable, but notably only in some cells, while the fusion protein remained cytosolic in others. Apparently, in land plants, the PTS2 of NgPKT was indeed functional but rather weak and inefficient in mediating peroxisome import for yet unknown reasons. A striking feature of the PTS2 of nearly all plant 3-ketoacyl-CoA thiolases is their atypical glutamine residue at position 2, replacing the normally hydrophobic residues (i.e., L, V or I; e.g., AtPKT3: RQx_5_HL). This residue is not only invariant in plant thiolases but also is very specific for thiolases. Hence, plant PEX7 might have evolved specific structural features for efficient recognition, import and possibly prioritization of plant thiolase based on its specific PTS2, and these plant PEX7 features might not be well compatible with the “atypical” PTS2 of NgPKT (RLx_5_HL). Future studies shall address whether NgPKT is efficiently imported into *N. oceanica* peroxisomes.

Contrary to NgMLS2 and NgPKT, the predicted PTS2 of NgHIT1 did not direct EYFP to peroxisomes. Consistent with the prediction of a bipartite ER/mitochondrial targeting signal by HECTAR (score: 0.80; Gschloessl et al., 2008), the punctate structures were indeed identified as mitochondria in both plant expression systems, using the presequence of *Saccharomyces cerevisiae* COXIV and CFP as mitochondrial marker (Fulda et al., 2002). Consistently, structural similarities between PTS2 and mitochondrial presequences have been reported (Osumi et al., 1992; Kunze et al., 2011; Kunze and Berger, 2015). Interestingly, NgHIT1-EYFP was dually targeted to both mitochondria and plastids (Figure 4). A second HIT homolog seemed non-peroxisomal (NgHIT2/3, EWM21969.1 and EWM28795.1, both w/o PTS2/PTS1). Peroxisome targeting of AtHIT3 had been validated by *in vivo* subcellular targeting analyses (Quan et al., 2010). *Arabidopsis* HINT4 showed a dual activity *in vitro*, acting on adenosine 5′-phosphosulfate both as a hydrolase (forming AMP) and as phosphorylase (forming ADP, Guranowski et al., 2010).

In addition to the predicted PTS2 proteins of *N. gaditana* that are homologs of *Arabidopsis* PTS2 proteins, nine proteins represent putative novel *Nannochloropsis* PTS2 proteins. In the protein annotated as “embryogenesis-associated protein EMB8” (EWM21473.1, Table 1) the predicted PTS2 (RLx_5_QL) was not conserved in closely related homologs and already mutated to a non-PTS2 in *N. salina* (RLx_5_RL). Nevertheless, the protein most likely is a true peroxisomal protein because the *N. gaditana* protein itself additionally possesses a predicted and conserved PTS1 (SRL>), which is also found in the homologs of *N. salina* (SRL>), *Ectocarpus* (ARL>), diatoms (*Phaeodactylum*, *Thalassiosira*, *Pseudo-nitzschia*, SRL>, *Fistulifera*, SKL>) but not in oomycetes. The PTS1 protein belongs to the functionally diverse superfamily of α/β hydrolases that includes diverse enzymes (proteases, lipases, peroxidases, esterases, epoxide hydrolases and dehalogenases), all characterized by a specific catalytic triad (Ser, Glu/Asp, and His). It will be interesting to study which physiological function this α/β hydrolase carries out in peroxisomes of stramenopiles.

The PTS2 of the remaining eight *Nannochloropsis* proteins, however, was hardly conserved in homologs outside of the genus *Nannochloropsis* (data not shown, e.g., vacuolar transporter chaperone 4, aldo/keto reductase, didehydrogluconate reductase; acetyl-/succinylornithine aminotransferase; two hypothetical proteins, Table 1). The data indicate that these PTS2 proteins are either specific to *Nannochloropsis* or represent false predictions.

The presence of peroxisomes in nearly all eukaryotes entails their vital importance in eukaryotes. The physiological significance of peroxisomes is further supported by evolution and maintenance of two conserved import pathways for soluble proteins to the peroxisomal matrix in most organisms. We concluded that *N. gaditana* maintained a fully functional PTS2 import pathway based on (i) the expression of an *N. gaditana PEX7* ortholog, (ii) the detection of 13 predicted PTS2 cargo proteins, (iii) our experimental validation of two proteins as true positives *in vivo* in diverse expression systems (NgMLS2 and NgPKT), and (iv) due to two additional PTS2 proteins predicted with high probability by complementary computational methodology (NgHIUase and NgEMB8). Due to single genes of these PTS2 cargo proteins in *Nannochloropsis*, this import pathway is crucial at least for two essential metabolic pathways of peroxisomes, namely fatty acid β-oxidation (NgPKT) and purine (urate) catabolism (NgHIUase), while the glyoxylate cycle might remain functional by activity of the PTS1-carrying MLS1 alone. The number of at least four probable PTS2 cargo proteins is considerable and exceeds, for instance, the number of only three PTS2 proteins in ascomycetes, such as *S. cerevisiae*.

To summarize the current knowledge about evolution and maintenance of the PTS1 and PTS2 import pathways in different taxa, we present a simplified phylogenetic tree focusing on stramenopiles (Figure 6). The PTS1 targeting pathway is present in all eukaryotes that possess peroxisomes. Generally, animals, fungi, and Viridiplantae (land plants, charophytes, and green algae) possess both PTS1/2 import pathways (Tolbert and Essner, 1981; Gabaldón, 2010) with a few exceptions due to secondary PTS2 pathway loss in specific animals (*C. elegans, Drosophila*, see section “Introduction”) and the rumen fungi, Neocallimasticalles and Microsporidia (Peraza-Reyes et al., 2010). The PTS2 import pathway is considered the more ancient import route for peroxisomal matrix proteins (Gabaldon et al., 2006; Bolte et al., 2015; Kunze and Berger, 2015; Reumann et al., 2016). By contrast, chromalveolates and particularly stramenopiles are amazingly heterogeneous with respect to both the presence of peroxisomes and the PTS2 import pathway. Non-coccidian apicomplexa (e.g., *Plasmodium*) lack peroxisomes entirely, while coccidian apicomplexa (e.g., *Toxoplasma*) possess solely the PTS1 import pathway (Schluter et al., 2006; Mbekeani et al., 2018), similar to nowadays red algae (Matsuzaki et al., 2004; Shinozaki et al., 2009). As shown by this study, some stramenopiles have maintained both pathways (*Eustigmatophyceae*, brown algae), while diatoms lost the PTS2 pathway entirely and transferred classical PTS2 cargo proteins (e.g., thiolase) to the PTS1 import pathway (Figure 6; Gonzalez et al., 2011). Because several classical PTS2 cargo proteins of animals, plants and amoebozoa (e.g., *Dictyostelium*) are PTS1 proteins in *Nannochloropsis* (e.g., citrate synthase, acyl-CoA oxidase, long-chain acyl-CoA synthetase, Supplementary Table 2), *Nannochloropsis* seems to follow the same evolutionary path of PTS2-to-PTS1 cargo conversion. Hence, PTS2-to-PTS1 cargo transition occurred in *Nannochloropsis* prior and independent of any mutational *PEX7* gene inactivation and while the PTS2 pathway is still fully functional. The driving force and evolutionary advantage for PTS2-to-PTS1 cargo transition remains elusive.

The absence of the PTS2 pathway in several groups of Chromalveolates can partly be explained by their evolutionary history. The organisms evolved undoubtedly by secondary endosymbiosis of a red alga, proven by the presence of chlorophyll c in their plastids (Cavalier-Smith, 1999). Recent publications support a polyphyletic origin of different subgroups of Chromalveolates rather than their common monophyletic origin because many nuclear, mitochondrial and plastidic genes were found to stem from unexpected origins, including even green algae, non-cyanobacterial prokaryotes and genes from sister groups of Chromalveolates (Yoon et al., 2002; Baurain et al., 2010; Keeling, 2010; Qiu et al., 2013; Dorrell et al., 2017). Moreover, Chromalveolates are now known to have gone through several independent endosymbiotic events. Hence, at least the two nuclear genomes, namely that of the heterotrophic host cell (which encoded all PEX and cargo proteins of both peroxisomal import pathways) and that of the red alga (which encoded all PEX and cargo proteins at least of the PTS1 pathway), were merged upon red alga engulfment in Chromalveolates. This new quasi-duplicated chimeric “nuclear genome entity” likely has allowed and favored the gradual reduction of PTS2 cargo numbers up to the complete loss of the PTS2 pathway for several reasons: (i) the evolutionary driving force for size reduction of the duplicated genome, (ii) the advantage that the red alga probably had already established and could contribute its (yet unknown) mechanism of PTS2-to-PTS1 cargo transition, and (iii) the simultaneous presence of two orthologous gene copies of peroxisomal enzymes, allowing PTS2-to-PTS1 cargo conversion of one copy via non-functional cytosolic enzyme intermediates (Reumann et al., 2016). The heterogeneity of the PTS2 pathway presence and its differential usage intensity in stramenopiles (Figure 6) support the hypothesis of an independent loss of the PTS2 pathway in red algae and several groups of Chromalveolates. In any case, the nuclear genome of the red alga definitely had a significant impact on genome enrichment and peroxisome biogenesis in ancestral Chromalveolates, which favored diversification, speciation and the amazing success of nowadays chromalveolates in conquering ecological niches.

## Data Availability Statement

The original contributions presented in the study are included in the article/Supplementary Material, further inquiries can be directed to the corresponding author.

## Author Contributions

DK and SR conceived and designed the research, and wrote the manuscript with contributions by IG. DK performed the computational analyses and cloned the *N. gaditana* genes. DK and PE performed the subcellular targeting analyses in plants. IG carried out *PEX7* expression and subcellular targeting analyses in *N. oceanica*. All authors read and approved the manuscript.

## Conflict of Interest

The authors declare that the research was conducted in the absence of any commercial or financial relationships that could be construed as a potential conflict of interest. The handling editor is currently organizing a Research Topic with one of the author SR.

## Footnotes

1 http://www.sb-roscoff.fr/hectar/

## Abbreviations

aa: amino acid
ALNS: allantoin synthase
ASW: artificial sea water
At: *Arabidopsis thaliana*
CaMV: cauliflower mosaic virus
DC: decarboxylase
DECR: 2,4-dienoyl-CoA reductase
DHNS: 1,4-dihydroxy-2-naphthoyl-CoA synthase
dpt: days post transformation
EMB8: embryogenesis-associated protein 8
EPA: eicosapentaenoic acid
EYFP/GFP: enhanced yellow/green fluorescent protein
HIT: histidine triad family protein
HIUase: 5-hydroxyisourate hydrolase
IndA: indigoidine synthase A
MDH: malate dehydrogenase
MLS: malate synthase
Ng: *Nannochloropsis gaditana*
OHCU: 2-oxo-4-hydroxy-4-carboxy-5-ureidoimidazoline
PEX: peroxin
PfkB: 6-phosphofructokinase
PGL3: 6-phosphogluconolactonase 3
PKT: peroxisomal 3-ketoacyl thiolase
PTS1/2: peroxisomal targeting signal type 1/2
PUFA: polyunsaturated fatty acid
PUKI: pseudouridine kinase
PUMY: pseudouridine monophosphate glycosylase
TEM: transmission electron microscopy.
