# Supplementary material for: Evolutionary Maintenance of the PTS2 Protein Import Pathway in the Stramenopile Alga Nannochloropsis
Source: Front Cell Dev Biol. 2020 Nov 19;8:593922. doi: 10.3389/fcell.2020.593922 (PMC7710942; doi:10.3389/fcell.2020.593922)
Supplement: Supplementary file 1 [file Data_Sheet_1.pdf]

Evolutionary Maintenance of the PTS2 Protein Import Pathway in the Stramenopile Alga *Nannochloropsis*

Journal: *Frontiers in Cell and Developmental Biology*;
Authors: Dmitry Kechasov, Imke de Grahl, Pierre Endries and Sigrun Reumann;
Affiliation: Plant Biochemistry and Infection Biology, Institute of Plant Science and Microbiology, Universität Hamburg, D-22609 Hamburg, Germany;
Email address of corresponding author: sigrun.reumann@uni-hamburg.de

Supplementary Figures

Suppl. Figure S1

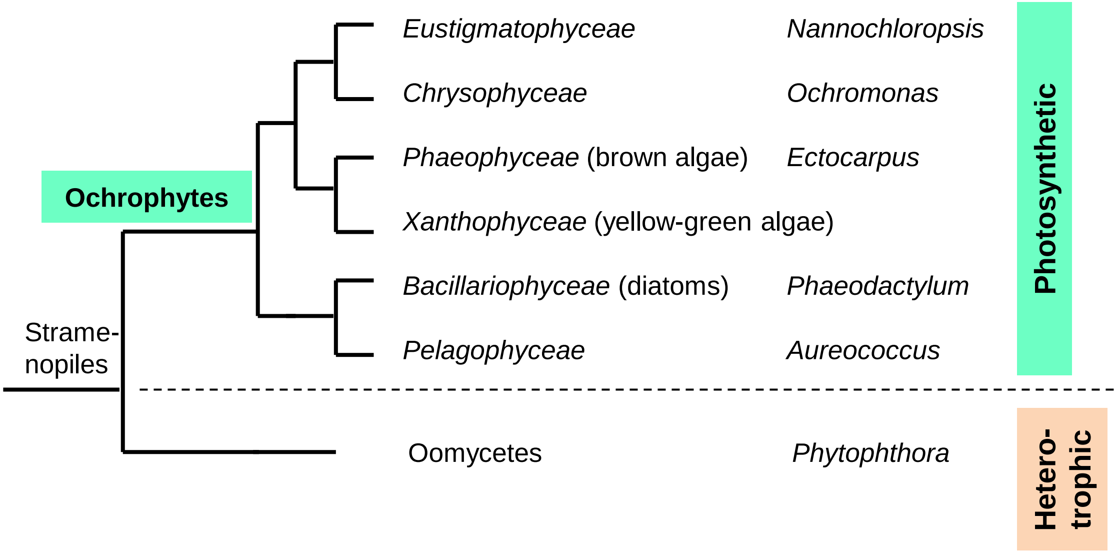

Suppl. Figure S1: Schematic phylogenetic tree of stramenopiles according to multiple gene analyses by Yoon et al. (2012). Stramenopiles contain both photosynthetic members (the ochrophytes), which possess complex plastids of red algal origin, and aplastidic and non-photosynthetic members (e.g. oomycetes). Ochrophytes include many ecologically important lineages (diatoms, kelps, pelagophytes) and *Nannochloropsis* as a potential model lineages for biofuels research. Ochrophytes form the most significant component of eukaryotic marine phytoplankton (Dorrel et al., 2017).

Suppl. Figure S2 A, B

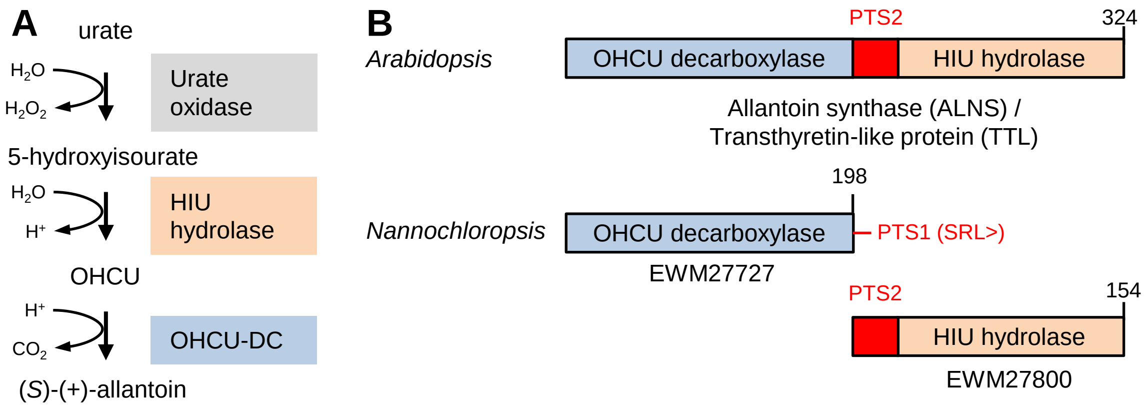

Suppl. Figure S2: Activity, domain architecture and evolution of peroxisomal enzymes of urate catabolism in *A. thaliana* and *N. gaditana*. (A) Urate degradation to (*S*)-(+)-allantoin is catalyzed by urate oxidase, 5-hydroxyisourate (HIU) hydrolase and (*S*)-2-oxo-4-hydroxy-4-carboxy-5-ureidoimidazoline (OHCU) decarboxylase (DC). **(B)** The domain architecture of OHCU-DC and HIU hydrolase shows for *A. thaliana* a bifunctional fusion protein with an unusual internal PTS2 and for *N. gaditana* two distinct PTS1- or PTS2-carrying enzymes. **(C)** and **(D)** are provided on the next page.

Suppl. Figure S2 C, D

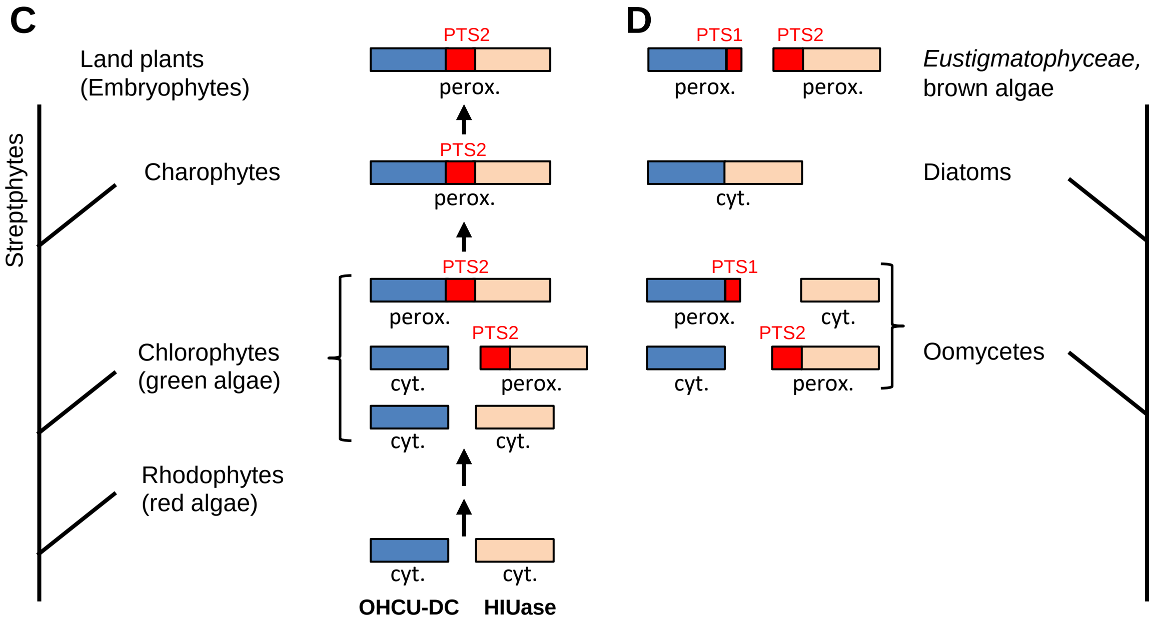

Suppl. Figure S2: Activity, domain architecture and evolution of peroxisomal enzymes of urate catabolism in *A. thaliana* and *N. gaditana*. (A) and **(B)** are shown on the previous page. **(C)** Schematic presentation of enzyme evolution in *Viridiplantae*. Initially both enzymes (OHCU-DC and HIUase) were separated and cytosolic, as still nowadays in Rhodophytes and few species of chlorophytes (*Trebouxiophyceae*). For instance, *Chlorella sorokiniana* still uses the ancient forms of single cytosolic enzymes. In the last common ancestor of Chlorophyceae and Streptophytes, HIUase first evolved a PTS2 (as found today in some *Trebouxiophyceae*). Examples include HIUase of *Micractinium conductrix* (RAx_5QL) and *Chlorella variabilis* (RAx_5QL). Subsequently, HIUase merged N-terminally with OHCU-DC, thereby leading to the unusual internal PTS2. Gene fusions (OHCU-DC-HIUase), all with the internal PTS2, are indeed detectable in two *Trebouxiophycean* species (*Trebouxia* sp. A1-2, *Coccomyxa subellipsoidea*, both RAx_5HL). **(D)** Schematic presentation of enzyme evolution in Stramenopiles with different variants in *Nannochloropsis* (*Eustigmatophyceae*, OHCU-DC with PTS1, HIUase with PTS2), diatoms (cytosolic protein fusion) and Oomycetes (separate enzymes with generally only one PTS-carrying enzyme). The constellation in Oomycetes implies peroxisome import of the other enzyme by oligomerization and piggy-back mechanism.

Suppl. Figure S3

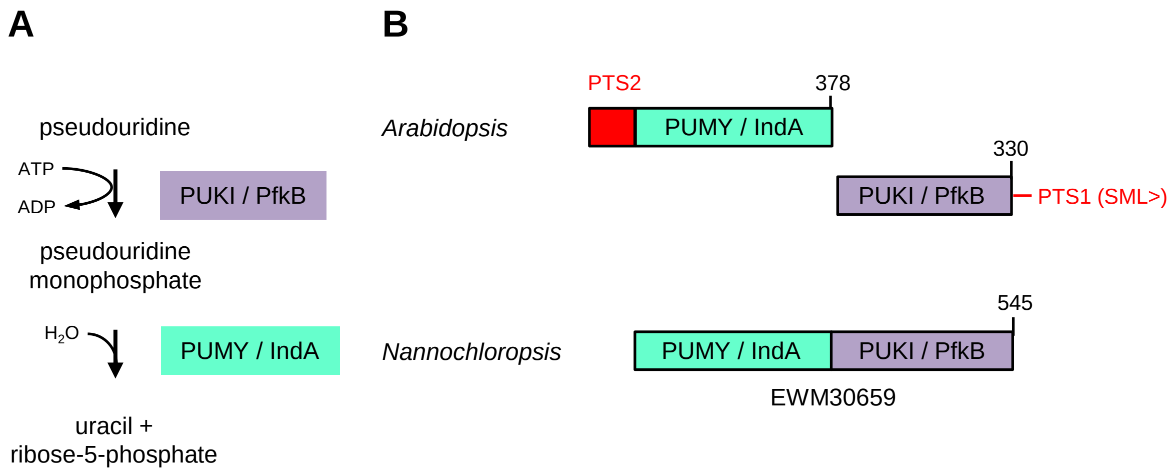

Suppl. Figure S3: Comparision of pseudouridine degradation catalyzed by single peroxisomal (*A. thaliana*) or a bifunctional cytosolic fusion enzyme (*N. gaditana*). (A) In *Arabidopsis*, pseudouridine degradation to uracil was recently demonstrated to be catalyzed by two peroxisomal enzymes, pseudouridine kinase (PUKI/PfkB) and pseudouridine monophosphate glycosylase (PUMY/IndA, Chen and Witte, 2020). **(B)** Comparative schematic diagram of the protein structures of PUKI/PfkB and PUMY/IndA in *A. thaliana* (two distinct PTS1- or PTS2-carrying enzymes) and *N. gaditana* (bifunctional fusion protein) without predicted PTS1/2 (but the decapeptide RLx_6HV).

Suppl. Figure S4

A: Thiolase (NgPKT)

```
N_gad_EWM24705     1 ------MSSTQTSQANKRLEHLSGHLVKKTSRPG
N_sal_TFJ86767     1 ------MSSTQTSQANKRLEHLSGHLVKKTSRPG
A_eut_KAF073646    1 ------------MQ---RIERIQQHLQ--APSSA
A_hyp_OQR92766     1 ------------MQ---RIERIQGHMR---PAAQ
G_spl_KAF132289    1 ------------MD---RIERIRSHVQQNKPSAA
A_lai_CCA20150     1 ---------------MERVRHLRSHLT----AKA
P_inf_KAF404366    1 ------------MD---RINRIRSHVANGSPAPA
A_ste_KAF069031    1 ------------MH---RIERIQQHLQ--APGSA
T_cla_QR95928    421 SLLHEPVPDVDNLPTMQRIERIQGHMH--APAA-
E_sp__CAB111896    1 ------------MEAARRMEVIGSHLS--ASSGA
H_fer_GBG28030     1 --------------MANRLEKLAAHLQ--VAGSA
C_roe_KAA016020    6 VVLTEGAEALRAHAAASRVEALARHLGSAAASGP
```

B: HIT1 (NgHIT1)

```
N_gad_EWM29206    1 MSQRLVRLSQHLLVH-
A_lai_CCA14803    1 --MRLIRFAEHATSS-
P_oli_TMW57389    1 MLHRLRVLNSHLAASS
```

C: HIUase (NgHIUase)

```
N_gad_EWM27800     1 -----------------MPAS-STTPATSRLTALAAHLSSV
P_bra_TYZ59927     1 -----------------M-----RGSADRRLASIQSHLQA-
E_sp__CAB110310    1 ----------------------MPNTPQERLDRITNHLAAG
G_spl_KAF133578    1 -----------------MPTSAPSPSSTRRISTLQQHLQM-
S_par_XP_012204    1 -----------------M-----N-----RLEVVAKHVMP-
S_dic_XP_008610    1 -----------------M-----N-----RLEVIAQHVMP-
P_cac_KAF179436  841 CSTGFNTWDLAVLVPNLLHSVTLMSSPSRRVNAVNRHLSS-
P_hal_XP_024577    1 --------------------------MASRINAVQRHASA-
A_ste_KAF071910    1 -----------------M----------ERLQTIQSHMAA-
A_eut_KAF074330    1 -----------------M----------ERLRRIQNHVAM-
```

Suppl. Figure S4: Analysis of PTS2 conservation of predicted *N. gaditana* PTS2 proteins in Stramenopiles. Putative orthologs of the PTS2-carrying thiolase (NgPKT, A), NgHIT1 (B) and HIUase (C) were identified by protein BLAST searches at NCBI using Genbank focusing on Stramenopiles. Homologs were aligned by COBALT (Papadopoulos and Agarwala, 2007) and sequence conservation labeled by Boxshade. Acronyms: A_eut, *Aphanomyces euteiches*; A_hyp, *Achlya hypogyna*; A_lai, *Albugo laibachii* Nc14; A_ste, *Aphanomyces stellatus*; C_roe, *Cafeteria roenbergensis*; E_sp, *Ectocarpus* sp. CCAP 1310/34; G_spl, *Globisporangium splendens*; H_fer, *Hondaea fermentalgiana*; N_gad, *Nannochloropsis gaditana* B-31; N_sal, *Nannochloropsis salina* CCMP1776; P_bra, *Pythium brassicum*; P_cac, *Phytophthora cactorum*; P_hal, *Plasmopara halstedii*; P_inf, *Phytophthora infestans*; P_oli, *Pythium oligandrum*; S_dic, *Saprolegnia diclina* VS20; S_par, *Saprolegnia parasitica* CBS 223.65; T_cla, *Thraustotheca clavata.*

Suppl. Figure S5 A

A

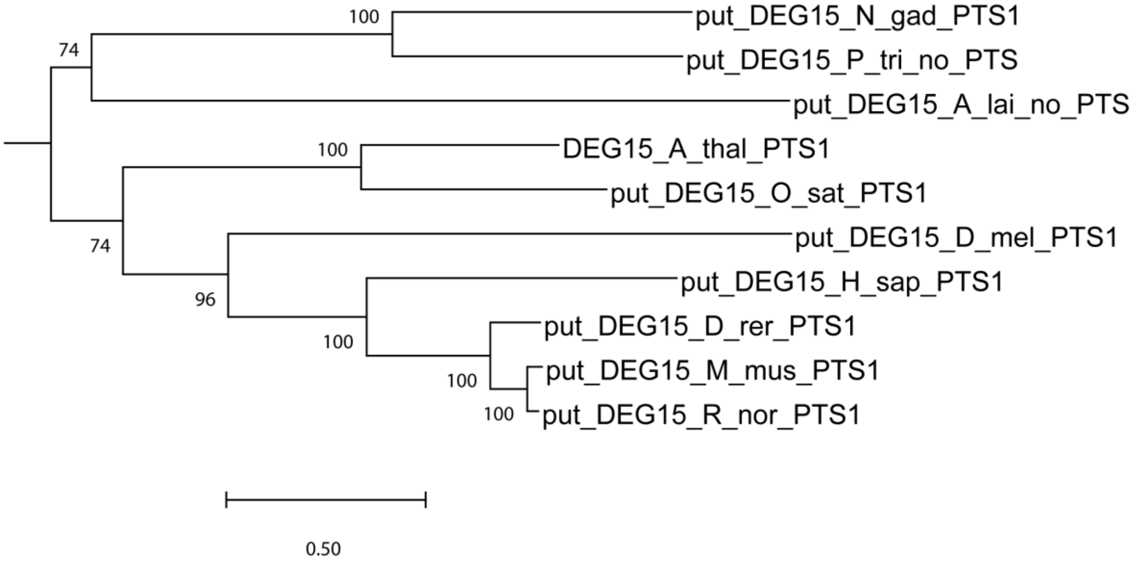

Suppl. Figure S5: Phylogenetic and homology analysis of DEG15 in *A. thaliana*, Stramenopiles and other eukaryotes. (A) Homologs of the PTS1-carrying DEG15 homolog of *N. gaditana* (putNgDEG15, EWM21659, SLL>) were identified by protein BLAST searches at NCBI using Genbank and focusing on Stramenopiles, green and red algae, land plants and animals. Sequences were aligned by MUSCLE (Edgar 2004) and the phylogenetic tree was constructed using the Bayesian inference method (Ronquist 2012). The branch support values were calculated as Bayesian posterior probabilities and are shown next to the branches. The platform Phylogeny.fr (Dereeper et al., 2008) was used for phylogenetic analysis, and MEGA X was used for tree visualization (Kumar, 2018). **(B)** is shown on the next page.

Suppl. Figure S5 B

B

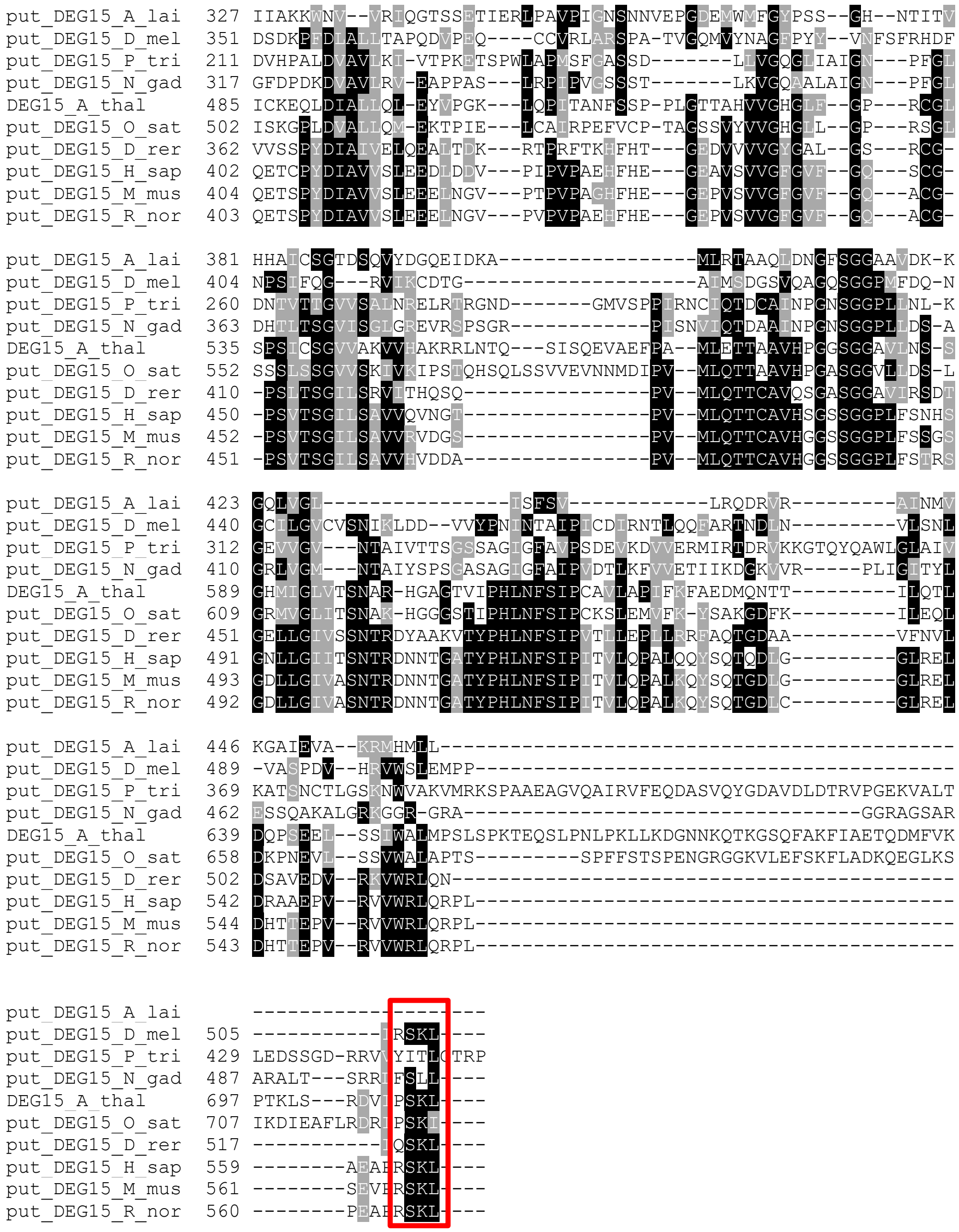

Suppl. Figure S5: Phylogenetic and homology analysis of DEG15 in *A. thaliana*, stramenopiles and other eukaryotes. (A) is shown on the previous page. **(B)** The alignment was performed with MUSCLE (v3.8.31, Edgar 2004). Only the conserved C-terminal domain is shown (amino acid residues 485 to 720 of AtDEG15). Acronyms: A_lai, *Albugo laibachii* Nc14 (CCA14173.1); A_thal, *Arabidopsis thaliana* (NP_174153.2); D_mel, *Drosophila melanogaster* (NP_611968.1); D_rer, *Danio rerio* (NP_001122182.1); H_sap, *Homo sapiens* (NP_775826.2); P_tri, *Phaeodactylum tricornutum* CCAP 1055/1 (XP_002183960.1); M_mus, *Mus musculus* (NP_082188.1); N_gad, *Nannochloropsis gaditana* B-31 (EWM21659.1); O_sat, *Oryza sativa* Japonica (XP_015640602.1); R_nor, *Rattus norvegicus* (NP_001102402.1).

Suppl. Figure S6

A. PEX7(NgPEX7, EWM28214.1):

MKRESFHAANTGGGGLGGPIPDAVFQTQFQCCS (intron 1) VEFSPFQEARLAVATAQYFGIIG
NGRQHILE (intron 2) IGPDGNLREIRSFLTQEGLYDCCWSEANQNQLVSASADGSLKLWDVMT
SDGYPVAH (intron 3) WQEHSAEVSSVHWNQVVKTNFLSASWDGSIKLWDPHHPTSLSTYCGHT
GCVYAGIHSPRHPHRFLSCGTDGSLRIWDTKLPPSHATSLGLGRAEGGAVQVVRAHEGEVLSADWDKY
QDFLVYTGGVDRSIKIWDLRRPSLPLGFLHGHGYAVRRLKTSPHQEGVLGSVSYDMSCRVWGPTRAGG
RAGELWRCEEHTEFVQGLDFHLFWPGRIATCGWDRRVCVWTLPLP

B. Malate synthase 2 (NgMLS2, EWM30341.1):

MFLSGSMAQ**RI**QVISR**HL**QQKKAFEEKNGATRRLTSFEAGPHI (intron 1) IEYDADVDGLHSS
IVDEVLTEGALSFLAELVQHFNKDVLE (intron 2)LYRRRAEVQTKIETGTYEFGFSPETAQIRK
(intron 3) GIWQVEPVPSVLLDRRVDVGDVAPDDARALVSALNSGAQGVQCDFDDGFCPTWRNV
LLGIRNVMDASQGVLCYPDSVRAHSVAIVSNAAVMMLRPRAWCMTEMHFYVNGRAIPGPLLDY(int-
ron 4) GLLIYHCGAELEMRGKGPFFYCSKVENYLEARLWNSIFTWSEVKLGLKK (intron 5)
NSVKACVLIENITASFQMDEILYELRNHSAGLNCGIWDYSASFIARFAQRSDMIFPDRTKYVTMECGF
MRNYMRLLVDTCHKRGAIATTGMAGLVLDPKWDKATRQAKLQEVRLAKHFEAEKGGSDGALIYDLNLK
GLVAEVFGGGLGRLNQLNRPLSSASIGPADLLEVPPGGITLEGVRFNTEIVVRFIDSWLRGRGSFVYR
NSAEDSATAEISRSQIWQWVRHGLYTEGKDAITLSLVMEFACETADELAQEGREAQIPPALVDDRVAS
ALSLYRMLVSVPVFPRFITTFLYEQALFHEFARRRSPIM

C. 3-Ketoacyl thiolase (NgPKT, EWM24705.1):

MSSTQTSQANK**RL**EHLSG**HL**VKK (intron 1) TSRPGFLGGDVVVVSALRTPL**C**KAKRGAFKSTT
TDDLLAPVLEAVVKQSGVDAATLGDIVVGNVLQPGSGAVGARMAQFYAGIPYQ (intron 2) VPL
CTLNRQCSSGLQAFLQVAASIQSGLYEAGIAGGVESMSLTDMSTSVPDVNFERVSENALSKDCTIPMG
QTSDEVATRFQVSREDQDRFAAASHAKAEAAVKAGKFAEEIVPVRVSSGGEDGGEDEVTVVREDEGIR
PGTTPEKLGGLRSSFSEGGSTTAGNSSQMTDGAAAVLVMSRGAATAQGMPVMGVLRGAAVVGVPPDIM
GIGPAVAIPAALAQARLRVEDVDVFEINEAFASQCLYCVRELKIPMEKVNPNGGAIALGHPLGATGAR
QIATLLHEMRRTRKRWGVVSMCIGTGMGAAAVFENEAAAN

D. Histidine triad family protein 1 (NgHIT1, EWM29206.1):

MSQ**RL**VRLSQ**HL**LVHRTSLHATSIAVRLSRTTRR**C**LVTGNQANTMADEVAESRKAAAKKMEEAADAGE
(intron 1) PTVFDKIVKKEIPSNFIYEDDDCVAFHDLSPQGPVHFLVIPKDRAGLSRLSKAEESH
KALLGHLLYVAQQVAKQEGLVPGGFRVVINDGPDGSQSVYHLHIHVIGGRQ (intron 2)MGWPPG

Suppl. Figure S6: Exon-intron structure of the PEX7 ortholog and three predicted PTS2 proteins from *N. gaditana* investigated experimentally in this study. For each *N. gaditana* protein, the Genbank accession number is provided for strain B-31 and intron positions are indicated. For the three PTS2 proteins investigated experimentally, the CDS of the first exon (underlined) was cloned from strain CCMP526 and placed upstream of the fluorescent reporter gene. The predicted PTS2 are highlighted in red and the most conserved four amino acid residues (e.g. RLx_5HL) are marked in bold. Cys residues of putative PTS2 cleavage sites are indicated (blue, C, D).

Suppl. Figure S7

```
P_inf         172 --------------------ETKAPVPMHKPMMQAAPVAAQQADTLLDLEAAKNLEAQQ---ASSEMART
P_tri         148 ----------------------------------------------------------------------
E_sil         221 --------------------------------------------EK------GVGEGQVGASARSL-VGQ
A_tha         267 VNGWATEFEQGQSQLMSSQMRS---------------------MDM------QNIAAME-QTRK-L-AHT
G_the          83 -------------EAWAEEFRR----------------------------------ERE-HGSE-L-SRA
C_ele         158 ----------------------------------------------------------------------
H_sap         209 LQHTA--------------------------------------------------------------SDF
N_oce_N-term  252 WSHAGQAF------PALEESH-----------------------DA------QATRAASGAVLEALREGG
N_oce_C-term    2 ----------------------------------------------------------------------
N_oce_full    252 WSHAGQAF------PALEESRVEAGSSSLPPSLEASLPPSLHEDDA------QATRAASGAVLEALREGG
N_gad           1 ----------------------------------------------------------------------
consensus     351

P_inf         219 MS--QNPNSKFQNSQFLKFMNQVSAGEVQIDEEKNEVVNGHLKMEGALEGAWEDTSD-MHSNRDL----F
P_tri         148 ----QMAHMVKQQQLMIRAQNQVSEHHHHRIRESNEQKSVSLDN-------WQNGVD-KEFGQQ------
E_sil         240 MA--ADP[illegible]GRFRDSELLRFATRLGTGGLRVSGDK--VVPGSGEAQG-LDEAWAGGASSQQQHQQAGDPAV
A_tha         307 LSQ--DGNPKFQNSRFLQFVSKMSRGELIIDENQ--VKQASAPGE------WATE----YEQQYL----G
G_the         103 LEETLNG[illegible]EKLAQSELRDFIAAVNDGSFQFDNGS--ILQPDGE---------------------------
C_ele         158 ----------------------------------------------------------------------
H_sap         217 VA--KVD[illegible]PKLANSEFLKFVRQIGEGQVSLESGA--GSGRA---QA------------------------
N_oce_N-term  287 RE--GGVCAKMARSEFVGFISQLNKGELAFEGNT--VI--------------CTTAE-ERRHQVAQGLGL
N_oce_C-term    2 -----------ARSEFVGFISQLNKGELAFEGNT--VVPRAGDFEG----AWAETAE-ERRHQVAQGLGL
N_oce_full    310 RE--GGVCAKMARSEFVGFISQLNKGELAFEGNT--VVPRAGDFEG----AWAETAE-ERRHQVAQGLGL
N_gad           1 ----------------------------------------------------------------------
consensus     421          k   sefl fm qv  g v  d     vv             w          q

P_inf         282 D-------------------------------------ASWKQS--------EN-----------G--SA
P_tri         200 --------------------------------------------------------------------VS
E_sil         305 AAAATKADFQVAYAEN--EAAEQQQQQGP-------LVGSFSDAWNGLDSGVGVREEPSL-KPSAAELLS
A_tha         359 PP------------SWADQFANEKLSHGP-----------------------------------------
G_the         144 ----------------------------------------------------------------------
C_ele         158 ---------------N--WADDFMEQQ-------------------------------------------
H_sap         256 --------------EQ--WAAEFIQQQ-------------------------------------------
N_oce_N-term  338 EG------------EW--EAAERAAVE-------------------------------------------
N_oce_C-term   54 EG------------EW--EAAERAAVEGPDIGAAAGLEGAWAEA--------HRVRGGQGADLSGS--VK
N_oce_full    371 EG------------EW--EAAERAAVEGPDIGAAAGLEGAWAEA--------HRVRGGQGADLSGS--VK
N_gad           1 ----------------------------------------------------------------------
consensus     491                    aae

P_inf         294 AA---ME--NAF-SEASAA-H-A----HPLEGAWKEAGTANA--------TSLDQAWG---------DSK
P_tri         202 DM---AP--VGMHEGVTQG-VSM----EELAAAWAEVADDNITV------G-H--------------DG-
E_sil         365 SA---QE--EAW-SDGKTG-L-APDFQEKMEAAWREVEQGGVGAGAGVGDPDLQAIWEESDD---DAAG-
A_tha         376 -EQWADEFASGR-GQQETA-----------EDQWV--NEFSK--------LNVD-DWI---------DEF
G_the         144 ----------------------------------------------------------------------
C_ele         168 ----------DN-YG--------------MENTWK-----------------------------------
H_sap         267 --------------G--------------TSDAWV------------------DQFTRPVNTSALDMEFE
N_oce_N-term      ----------------------------------------------------------------------
N_oce_C-term  100 AG---LD--EAW-AQAEVEAK-N----ADLESLWKEGAEGKD--------LDMDAFWDHVQSVAGEYDNA
N_oce_full    417 AG---LD--EAW-AQAEVEAK-N----ADLESLWKEGAEGKD--------LDMDAFWDHVQSVAGEYDNA
N_gad           1 ----------------------------------------------------------------------
consensus     561           a                  le  w                                 d

P_inf         335 TAEEKMMDSAWGESD-N-LEAIWEKAMAEAQ--TTDPFEDAWDNAT--NQDYTYKAENPFLDSSENFQKG
P_tri         240 --------LAQGATIEE-LAAAWAQAEAEYDSVDAATNLWNDTNDP--VYEFLNTEKPERVDQQDWMEQG
E_sil         423 --------------V-ETLDGVWSRTAATLE--A----GEGNL-----EAPYELSAENRFNDVDSPFEEG
A_tha         413 AEGPVGD--S--------SADAWANAYDEFLNEKNAGK---QTS-----GVYVFSDMNPYVGHPEPMKEG
G_the         144 ---------------------LWD----------PAGL---ETPF--SLRPYTFTPHNPFLGRPDCFRSG
C_ele         178 ------------DAQA--FEQRWEEIKRDMEKDESLQ----------SPENYVYQEANPFTTMSDPLMEG
H_sap         291 RAKSAIE--SDVDFWDK-LQAELEEMAKRDAEAHPWLSDYDDLTSATYDKGYQFEEENPLRDHPQPFEEG
N_oce_N-term      ----------------------------------------------------------------------
N_oce_C-term  151 MAKDAVPGISKADAV-E-LEASWQQQARDLEEWAARGAAEGGLEGG--QAAYGFADDNEFMDHADAFAEG
N_oce_full    468 MAKDAVPGISKADAV-E-LEASWQQQARDLEEWAARGAAEGGLEGG--QAAYGFADDNEFMDHADAFAEG
N_gad           1 ----------------------------------------------------------------------
consensus     631                   l a w     e                      y y e n fld  e f eg
```

Suppl. Figure S7: Analysis of *Nannochloropsis* PEX5 orthologs for the presence of the PEX7 binding domain in Stramenopiles. (Legend on next page).

Suppl. Figure S7 (cont.)

```
P_inf        399 IEFFKSGHLDDAILAFEAEVQQ-HPENSEAWRMLGECHAENDEDKSAIICLERAVEEDPYNLSALLALGV
P_tri        299 LREFNAGNLKEAVKAFEIELQYCNGDNSAAWKMLGRCHAENDMDREAIVCLEQAVDRDPYSPEALLLLGV
E_sil        467 VRLFEEGQIADAALCFEAEIAR-NPDNSQAWFMLGQSHAENDQDRLAISCLEKAVEIDPYSLDALLALGT
A_tha        465 QELFRKGLLSEAALALEAEVMK-NPENAEGWRLLGVTHAENDDDQQAIAAMMRAQEADPTNLEVLLALGV
G_the        178 VEMLQRGELVESVQALEAEVQE-HPDNCDAWLTLGLAHAENDEDVKAIIALNRAVQADPDNLDALLALGV
C_ele        224 DNLMRNGDIGNAMLAYEAAVQK-DPQDARAWCKLGLAHAENEKDQLAMQAFQKCLQIDAGNKEALLGLSV
H_sap        358 LRRLQEGDLPNAVLLFEAAVQQ-DPKHMEAWQYLGTTQAENEQELLAISALRRCLELKPDNQTALMALAV
N_oce_N-term     ----------------------------------------------------------------------
N_oce_C-term 217 MALFEAGELRQAVLAFEAEVQR-APHNAEAWRMLGMTHAENDEDKRAIACLERAVDHDPHSLPTLLALGV
N_oce_full   534 MALFEAGELRQAVLAFEAEVQR-APHNAEAWRMLGMTHAENDEDKRAIACLERAVDHDPHSLPTLLALGV
N_gad          1 --------------------------------------AENDEDKRAIACLERAVDQDPHSLPALLALGV
consensus    701 i lf  g l  ailafeaevq   p n eaw mlg  haendedk ai clerave dp  l allalgv


P_inf        468 SNVNELNPQGALKTLKAWVQHNPKFHGLEIQVDEY----SDG-----------------SLMDEVMQLML
P_tri        369 SYVNELNHAKALKNLKAWITHNPKFAGMELQVDMY----RDS------------LVDQESAFDEVQRLLV
E_sil        536 SYVNELDSQKALTNLKAWVEHNPKYSGLEIAVDEY----SDG-----------------TLMDEVMQLML
A_tha        534 SHTNELEQATALKYLYGWLRNHPKYGA--IAPPE----LADS-----------------LYHADIARLFN
G_the        247 SHTNELEQVNALTHLRSWITRHPEYSQLCPPQEEIGSLRETY-----------------TLHNEVTQIFT
C_ele        293 SQANEGMENEALHQLDKWMSSYLGSNSTQVTTTPP----LY-----------SSFLDS-DTFNRVEARFL
H_sap        427 SFTNESLQRQACETLRDWLRYTPAYAHLVTPAEEG----AGGAGLGPSKRILGSLLSD-SLFLEVKELFL
N_oce_N-term     ----------------------------------------------------------------------
N_oce_C-term 286 SYVNELDSVRALQNLKAWIEHNPKYQGLEIRVDEY----SDG-----------------SLMDEVMQLML
N_oce_full   603 SYVNELDSVRALQNLKAWIEHNPKYQGLEIRVDEY----SDG-----------------SLMDEVMQLML
N_gad         33 SYVNELDSVRALQNLKAWVEHNPKYQGLEIRVDEY----SDG-----------------SLMDEVTQLML
consensus    771 syvneld   al nlkawv hnpky glei vdey     dg                 sl dev qlml


P_inf        517 QARAHDP---------S--DS-DVQVVLGVLYNVSKDYDAAVSSFKAATDSQPDEYALWNKIGATLANSA
P_tri        423 QALEYDP---------V--DASDVLEAMGVVYNVSRDYVAAGGAFRRALDARPDDYQLWNKLGATLANGN
E_sil        585 QAQRWDA---------T--DA-DAHVVLGVLYNVSRDYDSAAEAFRRAIEARPNDHSLWNKLGATLANSR
A_tha        581 EASQLNP---------E--DA-DVHIVLGVLYNLSREFDRAITSFQTALQLKPNDYSLWNKLGATQANSV
G_the        300 RVLQTRP---------D--DV-ELHTVLGILYHLSYDYEKAIEHFREALRINPQDYSLWNKLGATLANFS
C_ele        347 DAARQQG---------ATPDP-DLQNALGVLYNLNRNFARAVDSLKLAISKNPTDARLWNRLGATLANGD
H_sap        492 AAVRLDP---------TSIDP-DVQCGLGVLFNLSGEYDKAVDCFTAALSVRPNDYLLWNKLGATLANGN
N_oce_N-term     ----------------------------------------------------------------------
N_oce_C-term 335 QAHDWDQATSGPGGQGS--DA-DVQIVLGVLYNVSHDFDSATAAFRQALLSRPSDYSLWNKLGATLANSQ
N_oce_full   652 QAHDWDQATSGPGGQGS--DA-DVQIVLGVLYNVSHDFDSATAAFRQALLSRPSDYSLWNKLGATLANSQ
N_gad         82 QAQDWDRATCGPEG--Q--DA-DVEVVLGVLYNVSHDYDSAAAAFRQALVARPSDYSLWNKLGATLANSQ
consensus    841 qa   d             da dv vvlgvlynvskdyd a   fr al  rp dyslwnklgatlans


P_inf        575 RSSEAIPAYHRALELKPRYARGWLNLGISHANLGNYEEATKCYLQALSLNNR---A-----------DHI
P_tri        482 QSQEALPAYHKALQLKPKYARAWLNMAISHSNLQNYDEAARCYLQTLSLNPA---A-----------IHC
E_sil        643 QSEAALPAYRRAIASKPGYARAWLNMGISQANLNRYEEASSCYLQALRLNPE---A-----------KHI
A_tha        639 QSADAISAYQQALDLKPNYVRAWANMGISYANQGMYKESIPYYVRALAMNPK---A-----------DNA
G_the        358 KSDEAVDAYIQALSIKPNYVRALANLGIAYSNQEMYEEGASCYLKALSINPG---A-----------SHI
C_ele        407 HTAEAISAYREALKLYPTYVRARYNLGISCMQLSSYDEALKHFLSALELQKGGNDA-----------SGI
H_sap        552 QSEEAVAAYRRALELQPGYIRSRYNLGISCINLGAHREAVEHFLEALNMQR---KSRGPRGEGGAMSENI
N_oce_N-term     ----------------------------------------------------------------------
N_oce_C-term 402 RSQEALPAYHRALDIKPKYARGWLNLGISHANLSSFDEAAKCYLQALRLNPE---A-----------MHI
N_oce_full   719 RSQEALPAYHRALDIKPKYARGWLNLGISHANLSSFDEAAKCYLQALRLNPE---A-----------MHI
N_gad        147 RSDEALPAYHRALDLKPKYARGWLNLGISHANLGRYEEAARCYLRALRLNPE---A-----------GHM
consensus    911 rs eaipayhralelkp yargwlnlgis anl  yeea kcyl al lnp    a            hi


P_inf        631 WSYLRICF--TCMERFDLVKIADTKDIARFREEFKLIDL----
P_tri        538 WSYLRIAL--SCSERWDLIQHAASQNLEAFKDLFDFVIYS---
E_sil        699 WSYLRIVF--SSMERFDLVQKAGKEDAGLFEDDFDLALPTPPP
A_tha        695 WQYLRLSL--SCASRQDMIEACESRNLDLLQKEFPL-------
G_the        414 WSSLRSVF--HFMDRSDLAARCDTENAEAFRGDIDF-------
C_ele        466 WTTMRSAAIRTSNVPDNLLRAVERRDLAAVKASLV--------
H_sap        619 WSTLRLAL--SMLGQSDAYGAADARDLSTLLTMFGLPQ-----
N_oce_N-term     -------------------------------------------
N_oce_C-term 458 WGYLRVTF--TSMERFDLVQLAGQQDPSVFDAEFGR-------
N_oce_full   775 WGYLRVTF--TSMERFDLVQLAGQQDPSVFDAEFGR-------
N_gad        203 WGYLRVTF--TSMERFDLVQLAGQQDPSLFEAEFGR-------
consensus    981 wsylri f  t merfdlv  a   d   f  ef
```

Suppl. Figure S7: Analysis of *Nannochloropsis* PEX5 orthologs for the presence of the PEX7 binding domain in Stramenopiles. The predicted genomic PEX5 CDS from *N. gaditana* (N_gad, EWM20982.1) and *N. oceanica* (626268) were aligned with PEX5 homologs from *A. thaliana* (A_tha, NP_200440), *H. sapiens* (H_sap, NP_001124495) and others by ClustalW (v2.1, at Phylogeny.fr). Sequence conservation was labelled by Boxshade. The alignment confirms that the four *Nannochloropsis* proteins are indeed PEX5 homologs and possess the conserved middle domain (boxed in red) that binds PEX7 in *Arabidopsis* and humans. The alignment of the weakly conserved N-terminal domain (approx. 260 aa of *A. thaliana* PEX5) is not shown.

Suppl. Figure S8

```
N_oce_mRNA    1 MPRPGGFPPQPGGPPPGGHGHKPGEPVPDAVFHTQ-FQCCSVEFSPFHEGRLAVATAQYFGIIGNGRQYI
N_gad         1 MKRESFHAANTGGGGLGG-------PIPDAVFQTQ-FQCCSVEFSPFQEARLAVATAQYFGIIGNGRQHI
A_tha         1 MP----------------------------VFKAP-FNGYSVKFSPFYESRLAVATAQNFGILGNGRIHV
H_sap         1 MSAVCGGAAR--------------------MLRTPGRHGYAAEFSPYLPGRLACATAQHYGIAGCG--TL
consensus     1 *..  . ... ..   ..       .........  ..  ...***. ..***.****..**.*.*....

N_oce_mRNA   70 LEMGPDGH-LRESRNFLTQEGLYDCSWSEVNENQLVSASADGSLKLWDTMTS-DDYPIAHWHEHQAEASS
N_gad        63 LEIGPDGN-LREIRSFLTQEGLYDCCWSEANQNQLVSASADGSLKLWDVMTS-DGYPVAHWQEHSAEVSS
A_tha        42 LELAPGAPGVTESVSYDTADAVYDVCWSESHDSVLIAAIGDGSVKIYDTALPPPSNPIRSFQEHAREVQS
H_sap        49 LILDPDEAGLRLFRSFDWNDGLFDVTWSENNEHVLITCSGDGSLQLWDTAKA--AGPLQVYKEHAQEVYS
consensus    71 *...*.. ........ ......* .*** ... *.....***....*. .. ...*.....**..*..*

N_oce_mRNA  138 VDWNLVGKNTFLSS-SWDGSIKHWDPTRPSSLATYLGHDGSCVYNAVWSPRHATRFLSCAADGSVRVWDA
N_gad       131 VHWNQVVKTNFLSA-SWDGSIKLWDPHHPTSLSTYCGHTG-CVYAGIHSPRHPHRFLSCGTDGSLRIWDT
A_tha       112 VDYNPTRRDSFLTS-SWDDTVKLWAMDRPASVRTFKEHAY-CVYQAVWNPKHGDVFASASGDCTLRIWDV
H_sap       117 VDWSQTRGEQLVVSGSWDQTVKLWDPTVGKSLCTFRGHES-IIYSTIWSPHIPGCFASASGDQTLRIWDV
consensus   141 *.... ... .... ***...*.*......*. *...*.. ..*.....*... .* * ..*...*.**.

N_oce_mRNA  207 SLPPSLPPAMGLGGGGMEGGMVQVVRAHEGEVLTADWDKYNEWVVYSGGVDRSVKIWDLRQPSLPLSFLH
N_gad       199 KLPPSHATSLGLG--RAEGGAVQVVRAHEGEVLSADWDKYQDFLVYTGGVDRSIKIWDLRRPSLPLGFLH
A_tha       180 REP----------------GSTMIIPAHDFEILSCDWNKYDDCILATSSVDKTVKVWDVRSYRVPLAVLN
H_sap       186 KAA----------------GVRIVIPAHQAEILSCDWCKYNENLLVTGAVDCSLRGWDLRNVRQPVFELL
consensus   211 .....    ....    ..*..... **..*.*. **.**.........**.....**.*.. .*...*.

N_oce_mRNA  277 GHGFAVRRLKASPHQGELIGSVSYDMSFRLWGAAREGGRVGEVWRCEYHTEFVQGLDFDLFMPGRIATCS
N_gad       267 GHGYAVRRLKTSPHQEGVLGSVSYDMSCRVWGPTRAGGRAGELWRCEEHTEFVQGLDFHLFWPGRIATCG
A_tha       234 GHGYAVRKVKFSPHRRSLIASCSYDMSVCLWDYMVEDALVG---RYDHHTEFAVGIDMSVLVEGLMASTG
H_sap       240 GHTYAIRRVKFSPFHASVLASCSYDFTVRFWNFSKPDSLLET---VEHHTEFTCGLDFSLQSPTQVADCS
consensus   281 **..*.*..*.**.......* ***.....*..... . .........****..*.*.........*..

N_oce_mRNA  347 WDRRVCVWRMPFL----
N_gad       337 WDRRVCVWTLPLP----
A_tha       301 WDELVYVWQQGMDPRAS
H_sap       307 WDETIKIYDPACLTIPA
consensus   351 ** ..... ....
```

Suppl. Figure S8: Homology analysis of two putative PEX7 homologs from *Nannochloropsis* by multiple sequence alignment with *Arabidopsis* and human PEX7. The translated CDS of a full-length mRNA of a putative PEX7 homolog from *N. oceanica* CCMP1779 (N_oce_mRNA, 583661) was aligned with the predicted PEX7 from *N. gaditana* (N_gad, EWM28214.1) and with PEX7 from *A. thaliana* (A_tha, NP_174220) and *H. sapiens* (H_sap, NP_000279) by ClustalW (v2.1, at Phylogeny.fr). Sequence conservation was labelled by Boxshade. The alignment confirms that both *Nannochloropsis* proteins are indeed PEX7 homologs of *Arabidopsis* and human PEX7 and that *N. oceanica* expresses a full-length PEX7 mRNA.

Suppl. Figure S9

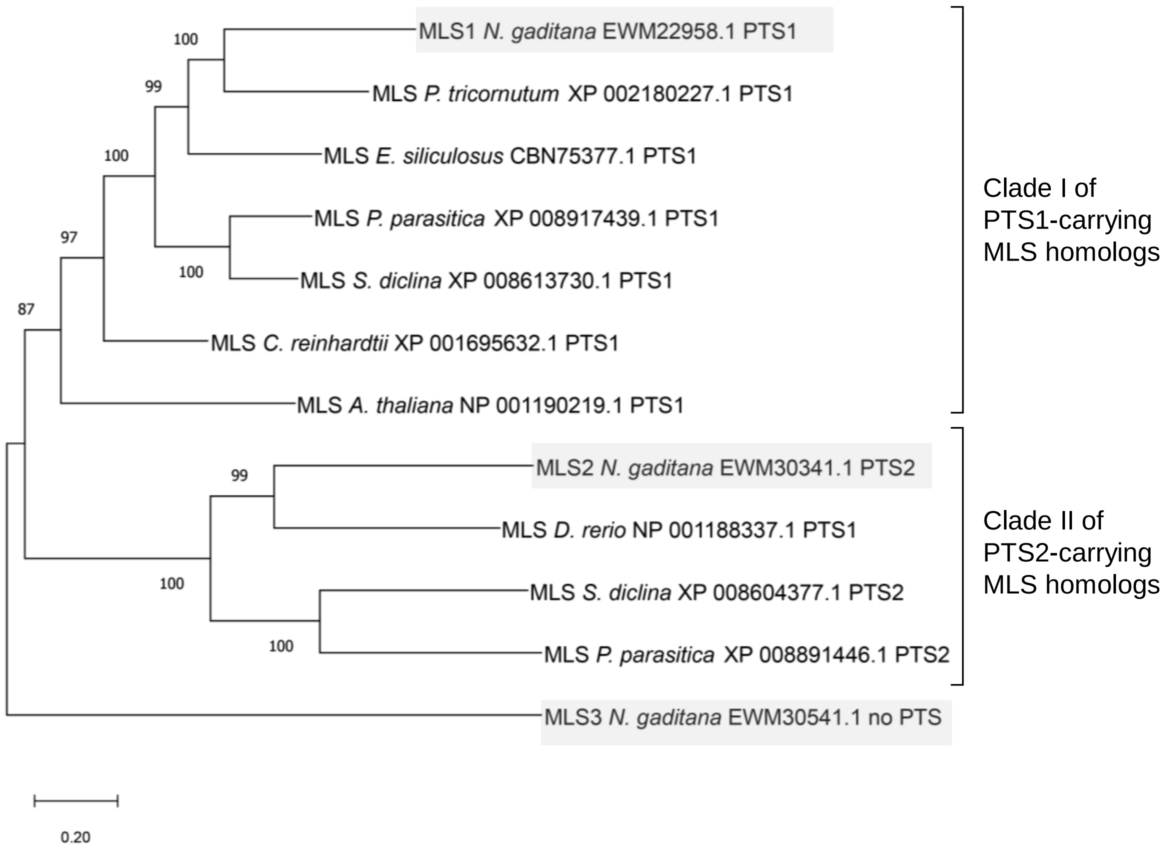

Suppl. Figure S9: Phylogenetic analysis of peroxisomal MLS isoforms from *N. gaditana*. Homologs of the PTS1- and PTS2-carrying MLS isoforms of *N. gaditana* (NgMLS1, EWM22958.1, SRL>; NgMLS2, EWM30341.1, RLx$_5$HL) were identified by protein BLAST searches at NCBI using Genbank and focusing on Stramenopiles, green and red algae, land plants and animals. Multiple sequence alignment of homologous proteins was performed in MUSCLE (Edgar 2004) and the proteins were analyzed for predicted PTS1 using the PredPlantPTS1 prediction server (http://ppp.gobics.de) and for predicted PTS2 using a manual motif search algorithm. The phylogenetic tree was constructed using the Bayesian inference method (Ronquist 2012). The branch support values were calculated as Bayesian posterior probabilities and are shown next to the branches. The platform Phylogeny.fr (Dereeper et al., 2008) was used for phylogenetic analysis, and the tree was visualized in MEGA X (Kumar, 2018). Among selected organisms, only Nannochloropsis and two oomycete species (*Saprolegnia diclina* VS20 and *Phytophthora parasitica*) had both, a predicted PTS1- and a PTS2-carrying MLS isoforms. The phylogenetic analysis revealed two distinct clades of PTS1-carrying and PTS2-carrying MLS isoforms (except for *D. rerio*). The non-peroxisomal MLS isoform of *N. gaditana* was used as an out-group.
