# Supplementary material for: Evolutionary Maintenance of the PTS2 Protein Import Pathway in the Stramenopile Alga Nannochloropsis
Source: Front Cell Dev Biol. 2020 Nov 19;8:593922. doi: 10.3389/fcell.2020.593922 (PMC7710942; doi:10.3389/fcell.2020.593922)
Supplement: Supplementary file 2 [file Data_Sheet_2.pdf]

Evolutionary Maintenance of the PTS2 Protein Import Pathway in the Stramenopile Alga *Nannochloropsis*

Journal: *Frontiers in Cell and Developmental Biology*;
Authors: Dmitry Kechasov, Imke de Grahl, Pierre Endries and Sigrun Reumann;
Affiliation: Plant Biochemistry and Infection Biology, Institute of Plant Science and Microbiology, Universität Hamburg, D-22609 Hamburg, Germany;
Email address of corresponding author: sigrun.reumann@uni-hamburg.de

SUPPLEMENTARY TABLES

Table S1: Presence of PEX proteins in *A. thaliana* compared to *N. gaditana* B-31 and *P. tricornutum*. The prediction of the PEX proteins was performed by homology search (BLASTp, NCBI, BLOSUM62 matrix and standard parameters) using the PEX proteins of *A. thaliana* as queries (Cross et al., 2016) against *N. gaditana* strain B-31 and *P. tricornutum* in the non-redundant protein database. Proteins with significant similarity (E-value < 10^{-3}, >40% identity, >60% of query length) were considered homologs. The *Arabidopsis* PEX proteins involved in PTS2 protein transport are marked in bold. Their homologs were found in *Nannochloropsis* (this study) but not in *Phaeodactylum*, as reported by Gonzalez et al. (2011). N.d.: not detected

Protein	Function	*A. thaliana*	*N. gaditana* B-31	*P. tricornutum*
			Accession number	
PEX1	AAA-ATPase complex	At5g08470	EWM21759.1	XP_002181897.1
PEX2	Ubiquitin ligase of RING complex	At5g62810.1	EWM25424.1	XP_002184009.1
PEX3	Membrane protein import	At3g18160.1; At1g48635.2	EWM25801.1	XP_002185591.1
PEX4	Ubiquitin-conjugating enzyme (E2 ligase)	At5g25760.1	EWM29846.1	XP_002182000.1
PEX5	Receptor for PTS1 proteins	At5g56290.1	EWM20982.1	XP_002177392.1
PEX6	AAA-ATPase complex	At1g03000.1	EWM26487.1	XP_002180890.1
**PEX7**	**Receptor for PTS2 proteins**	**At1g29260.1**	**EWM28214.1**	**n.d.**
PEX10	Ubiquitin ligase of RING complex	At2g26350.1	EWM23598.1	XP_002181643.1
PEX11a; PEX11b; PEX11c; PEX11d; PEX11e	Perox. division/ proliferation	At1g47750.1; At3g47430.1; At2g45740.1; At3g61070.1; At1g01820.1	EWM23691.1	XP_002186458.1
PEX12	Ubiquitin ligase of RING complex	At3g04460.1	EWM24718.1	XP_002184072.1
**PEX13**	**Receptor (PEX5/PEX7) docking**	**At3g07560.1**	**EWM21386.1**	**n.d.**
**PEX14**	**Receptor (PEX5/PEX7) docking**	**At5g62810.1**	**EWM25114.1**	**n.d.**
PEX16	Membrane protein import	At2g45690.1	EWM26598.1	n.d.
PEX19	Membrane protein import	At3g03490.1; At5g17550.1	EWM30185.1	XP_002176754.1
PEX22	Membrane anchor of PEX4	At3g21865.1	n.d.	n.d.
PEX26 (APEM9)	Membrane anchor for AAA complex	At3g10572.1	n.d.	n.d.

Table S2: PTS analysis of peroxisomal homologs of known *A. thaliana* PTS2 proteins in *N. gaditana*. Known *A. thaliana* PTS2 proteins were used as queries for homology search (BLASTp, BLOSUM62 matrix and standard parameters) to detect the corresponding orthologs in *N. gaditana* B-31. PTS1 were predicted by the prediction server, PredPlantPTS1 (ppp.gobics.de), while PTS2 were predicted manually. Only putative peroxisomal orthologs are listed. Acronyms: ACD, alpha-crystallin domain; ACX, acyl-CoA oxidase; ALNS, allantoin synthase; ASP, aspartate aminotransferase; CSY, citrate synthase; DHNS, 1,4-dihydroxy-2-naphthoyl-CoA synthase; HIT, histidine triad family protein; HINT3, histidine triad nucleotide-binding protein 3; IndA, indigoidine synthase A; LACS, long-chain acyl-CoA synthetase; OHCU DC, 2-oxo-4-hydroxy-4-carboxy-5-ureidoimidazoline decarboxylase; PfkB, 6-phosphofructokinase; pMDH, peroxisomal malate dehydrogenase; PKT, 3-keto-acyl-CoA thiolase; PUMY, pseudouridine monophosphate glycosylase; PUKI, pseudouridine kinase; TTL, transthyretin-like protein.

A. thaliana		***N. gaditana* B-31**	
**Acc. number**	**Acronym**	**Acc. number**	**PTS1/2 (peptide)**
At5g65110.1	ACX2	EWM23417<br>EWM30374	PTS1 (ARL>)<br>PTS1 (ARL>)
At1g06290.1	ACX3		
At1g06310.1	ACX6		
At3g58740.1	CSY1	EWM23670	PTS1 (ARL>)
At3g58750.1	CSY2		
At2g42790.1	CSY3		
At5g11520.1	ASP3	EWM21138	PTS1 (AHL>)
		EWM27204	no PTS1/2
At3g05970.1	LACS6	EWM20588	PTS1 (ARL>)
At5g48880.1	KAT5/PKT1/2	EWM24705	PTS2 (RLx_5HL)
At2g33150.1	PKT3		
At1g04710.1	PKT4		
At2g22780.1	pMDH1	EWM27487	no PTS1/2<br>(*N. oceanica*: SHL>)
At5g09660.1	pMDH2		
At5g58220.1	TTL/ALNS	EWM27800<br>(Transthyretin)<br>EWM27727<br>(OHCU DC)	PTS2 (RLx_5HL)<br><br>PTS1 (SRL>)
At1g50510.1	IndA/PUMY	EWM30659 (fusion protein with C-terminal PfkB/PUKI)	(PTS2: RLx_6HV?)
At1g60550.1	DHNS	EWM27835	no PTS1/2<br>(but PTS1 in *N. salina*, *Ectocarpus* and diatoms)
At3g56490.1	HIT3	EWM29205/6	PTS2 (RLx_5HL)
At5g48545.1	HINT3	EWM28795	no PTS1/2
At1g06460.1	ACD32.1	EWM22022	no PTS1/2

Table S3: Oligonucleotide primers used in this study

Oligonucleotide primers are listed that were used for expression analyses of *NgPEX7* or gene cloning from *N. gaditana* CCMP526 for subcellular localization studies. Accession numbers are provided for both *N. gaditana* B-31 (e.g. EWM28214.1) and CCMP526 (e.g. NGA_0680400). Restriction sites are underlined. The DNA corresponding to the N-terminal exons of the *N. gaditana* CCMP526 proteins were subcloned as C-terminal fusions with EYFP into the plant expression vector pCAT (NgMLS2-EYFP, NgPKT-EYFP, and NgHIT1-EYFP) and/or as C-terminal fusions with mVenus into the *N. oceanica* expression vector pNoc ox Venus (NgHIT1-Venus and NgMLS2-Venus). The full-length CDS of AtpMDH1 (At2g22780) was subcloned likewise into pNoc ox Venus and used as a known Arabidopsis PTS2 protein in *N. oceanica*.

**Gene acronym (Acc. numbers for B-31; CCMP526)**	**Sequence (5' to 3‘, restriction sites underlined)**	**Application**
NgPEX7	fw1: TCCCAGAGTTTGATGCTGCCG	Expression analysis
(EWM28214.1;	fw2: ATGAAGCGAGAATCCTTCC	
NGA_0680400)	fw3: CAGGCACCGTCCAAATCG	
	rv1: GCTTTTTAACGCAGGAAGGC	
	rv2: TTCCTACGGAAGGGGGAG	
	rv3: CGCTCTGCGGTGCTTTTG	
NgMLS2	fw: CACCATGGGATTCTTGTCTGGCAGCATG	Cloning into pCAT
(EWM30341.1;	rv: TGCCGCGGTGATATGTGGCCCAGCTTC	
NGA_0373902)	fw: AAGGCGCGCCATGTTCTTGTCTGGCAGCATGGCG	Cloning into
	rv: AAACAATTGGATATGTGGCCCAGCTTCAAATG	pNoc ox Venus
NgPKT	fw: CACCATGGGAAGCAGCACACAAACTTCG	Cloning into pCAT
(EWM24705.1;	rv: TATCCGCGGCCTTTTTGACCAGATGTCC	
NGA_0171000)		
NgHIT1	fw: CAGGAGCTCTCATGAGCCAACGTCTTGTCCGCCTCTCTC	Cloning into pCAT
(EWM29206.1)	rv: TGCCGCGGACTCGCCCGCATCCGC	
	fw: AAGGCGCGCCATGAGCCAACGTCTTGTCCGCCTC	Cloning into
	rv: AAACAATTGCTCGCCCGCATCCGCTGCCTC	pNoc ox Venus
AtpMDH1	fw: AAAGGCGCGCCATGGATCCAAACCAACGTATC	Cloning into
(At2g22780)	rv: TTTGAATTCTTTCTTCGCAAAGGTAACACC	pNoc ox Venus

Peroxisomal marker mCer-PTS1 for *N. oceanica*	fw: CAGGAGGGCCCATGCCCCTCTCGCAG<br>rv: AAGCGGCCGCGGCCTTTTGCTGGCCTTTTGCTCAC	Replacement of the hygromycin resistance casette of pNoc ox *Venus* with that of blasticidin
	fw: AAGGCGCGCCATGGTGAGCAAGGGCGAGGAGCTG<br>rv: AAGAGCTCTTCAGAGCTTCGAAACAGGCTTGTAC	Replacement of *Venus* against *mCerulean* extended by SKL>
